# An analytical workflow enabling selective enrichment of proteoglycans and heparin ligands from the human aorta

**DOI:** 10.1016/j.mbplus.2026.100207

**Published:** 2026-11-01

**Authors:** Alexander F. Minns, Ren Jie Tuieng, Marsioleda Kemberi, Yousuf Salmasi, Thanos Athanasiou, Anthony D. Whetton, Alexander Eckersley, Michael J. Sherratt, Salvatore Santamaria

**Affiliations:** a Discipline of Clinical Sciences, School of Biosciences, Faculty of Health and Medical Sciences, University of Surrey, Guildford, Surrey, United Kingdom; b Department of Immunology and Inflammation, Imperial College London, London, United Kingdom; c Division of Cell Matrix Biology & Regenerative Medicine, School of Biological Sciences, Faculty of Biology, Medicine and Health, The University of Manchester, Manchester, United Kingdom; d Singapore Nuclear Research and Safety Institute, National University of Singapore, Singapore; e Barts Health NHS Trust, The Royal Hospital, London, United Kingdom; f Department of Surgery and Cancer, Faculty of Medicine, Imperial College London, London, United Kingdom; g Veterinary Health Innovation Engine, Schools of Veterinary Medicine and Biosciences, University of Surrey, Guildford, Surrey, United Kingdom; h Division of Musculoskeletal & Dermatological Sciences, School of Biological Science, Faculty of Biology, Medicine and Health, The University of Manchester, Manchester, United Kingdom; i Manchester Cell Matrix Centre, School of Biological Science, Faculty of Biology, Medicine and Health, The University of Manchester, Manchester, United Kingdom

**Keywords:** Proteoglycans, Matrisome, Secreted factors, Proteomics, Thoracic aortic aneurysms

## Abstract

Proteoglycans, extracellular matrix (ECM) regulators and secreted factors present distinct challenges to detection and analysis by mass spectrometry and downstream biochemical characterization. These include the presence of glycosaminoglycans in proteoglycans and low abundance/complex post-translational regulation in the case of ECM regulators and secreted factors. As a result, our understanding of diseases where dysregulated ECM remodeling is a major pathogenic driver, like thoracic aortic aneurysms (TAA), remains limited. Characterization of aortic proteoglycans and matrisome-associated proteins is fundamental for the identification of candidate biomarkers and therapeutic targets in TAA.

Here, we present an analytical workflow enabling selective enrichment of proteoglycans and ECM regulators/secreted factors from human TAA aortas, based on their different isoelectric points. Anion exchange (AEX) and cation exchange (CEX) chromatography were sequentially employed using diethylaminoethyl and heparin Sepharose, respectively, before analysis by liquid chromatography-tandem mass spectrometry to selectively enrich in proteoglycans and ECM regulators/secreted factors, respectively. We have identified 1202 proteins in protein extracts, 1138 in AEX early peaks (eluted at 250–600 mM NaCl) and 1146 in AEX late peaks (eluted at 650–900 mM NaCl). When ranked by overall abundance, 4 out of the top 10 most abundant proteins in AEX late peaks were proteoglycans compared to only one proteoglycan in protein extracts. In CEX peaks, 187 matrisome proteins were identified, including 53 ECM regulators and 23 secreted factors, as well as 1016 non-ECM proteins. Selective enrichment was orthogonally validated by immunoblot analysis. Our workflow is readily translatable to other connective tissues for in-depth proteoglycanome/heparin ligandome profiling.

## Highlights

• Proteoglycans, ECM regulators and secreted factors are challenging to detect.
• We describe a pipeline based on the selective enrichment of these matrisome classes.
• Our pipeline is based on the differential isoelectric point.
• We applied our approach to human thoracic aortic aneurysm samples.
• In addition to the proteoglycanome and proteome, we uncovered the heparin ligandome.

## Introduction

The aorta is the largest artery in the human body, transporting oxygenated blood directly from the heart to the rest of the circulatory system. It consists of 3 distinct layers (from the lumen: tunica intima, media and adventitia), all relatively rich in extracellular matrix (ECM) compared to smaller vessels. The key ECM components of the aortic wall responsible for its biomechanical properties are collagens, elastic fibers (elastin and associated microfibrils) and proteoglycans, which are all proteolytically remodeled at different stages of several vascular diseases including aortic aneurysms and atherosclerosis [1].

Proteoglycans have long chains of repeating disaccharide units called glycosaminoglycans (GAGs) covalently attached to their protein core. Proteoglycans are classified by cellular/extracellular location, presence of specific domains, sequence homology, and by the type of covalently-attached GAG: chondroitin sulfate (CS)/dermatan sulfate, keratan sulphate, or heparan sulfate [2]. Classification solely by GAG types is hindered, however, by the ability of proteoglycans to carry different types of GAGs in different tissues and cell types [3]. So far, around 90 proteoglycans have been experimentally identified, including pericellular, transmembrane and intracellular proteins [4], and this list is continually growing. Two major groups of aortic proteoglycans include the large aggregating/hyaluronan-binding/hyalectans (aggrecan and versican) and the small leucine-rich proteoglycans (SLRPs, including biglycan, decorin and lumican).

Aggrecan and versican accumulate in thoracic aortic aneurysms (TAA), localized enlargements of the thoracic aorta, causing local biomechanical stress which predisposes to fatal dissections [5], [6]. In humans, loss-of- function mutations in biglycan cause syndromic forms of TAA and dissections (TAAD) [7]. Decreased levels of decorin have been associated with TAAD both in mouse models and humans [8], [9]. Characterizing proteoglycans and their fragments is therefore instrumental in understanding the pathogenetic mechanisms underlying TAA and identifying candidate biomarkers or potential therapeutic targets.

In contrast to purified recombinant proteoglycans [10], [11], analysis of tissue proteoglycans by liquid chromatography tandem mass spectrometry (LC-MS/MS) is negatively affected by high GAG density, endogenous proteolysis and high affinity interactions with other ECM components, resulting in limited sequence coverage, in particular for hyalectans [3], [12].

Methods to analyze proteoglycans from cell cultures were initially described by the Hascall group in the 1980s and involved extraction in guanidine hydrochloride (Gu-HCl) as a chaotropic agent, followed by isolation through anion exchange (AEX) chromatography [13], [14]. Recently, AEX, which is based on the selective binding of negatively charged GAGs to a positively charged matrix, has been combined with high resolution LC-MS/MS to improve detection of TAA proteoglycans [5]. However, while efficient in isolating proteoglycans from a variety of tissue and cellular sources [15], [16], the AEX approach alone does not robustly identify ECM regulators (a class of matrisome-associated proteins which includes proteases and their inhibitors) or secreted factors (cytokines, growth factors and morphogens) [17], which are presumably extracted together with proteoglycans under chaotropic conditions. Analysis of ECM regulators and secreted factors is further hampered by complex post-translational modifications, low abundance, and association with sulfated GAGs within the ECM [18], [19], therefore often requiring specific, targeted methods, such as chemical probing [20].

Here, we present a workflow designed to specifically characterize proteoglycans, ECM regulators and secreted factors from the human aorta, based on their different isoelectric points (pIs). We used tissues from patients undergoing elective TAA repair surgery, due to the quantity of tissue available, and the mechanistic and pathological implications of proteoglycans, ECM regulators and secreted factors in TAA [5], [21], [22].

## Results

### Workflow overview

We hypothesized that, compared to proteoglycans, which are, by definition, decorated with negatively charged GAGs, most ECM regulators, including proteases and their inhibitors, and secreted factors, have a higher pI (overall less negative charge) which may allow their binding to GAGs and other ECM components. Proteins from human TAA tissues were extracted under chaotropic conditions and sequentially subjected to AEX and cation exchange (CEX) chromatography to selectively enrich the proteome in proteoglycans and ECM regulators/secreted factors, respectively (Fig. 1). Peptide/protein coverage was analyzed by LC-MS/MS followed by Peptide Location Fingerprinting (PLF), an analytical approach designed to observe regional variations in mass spectrometric peptide coverage along the protein sequence [23], [24], [25].

**Fig. 1:**
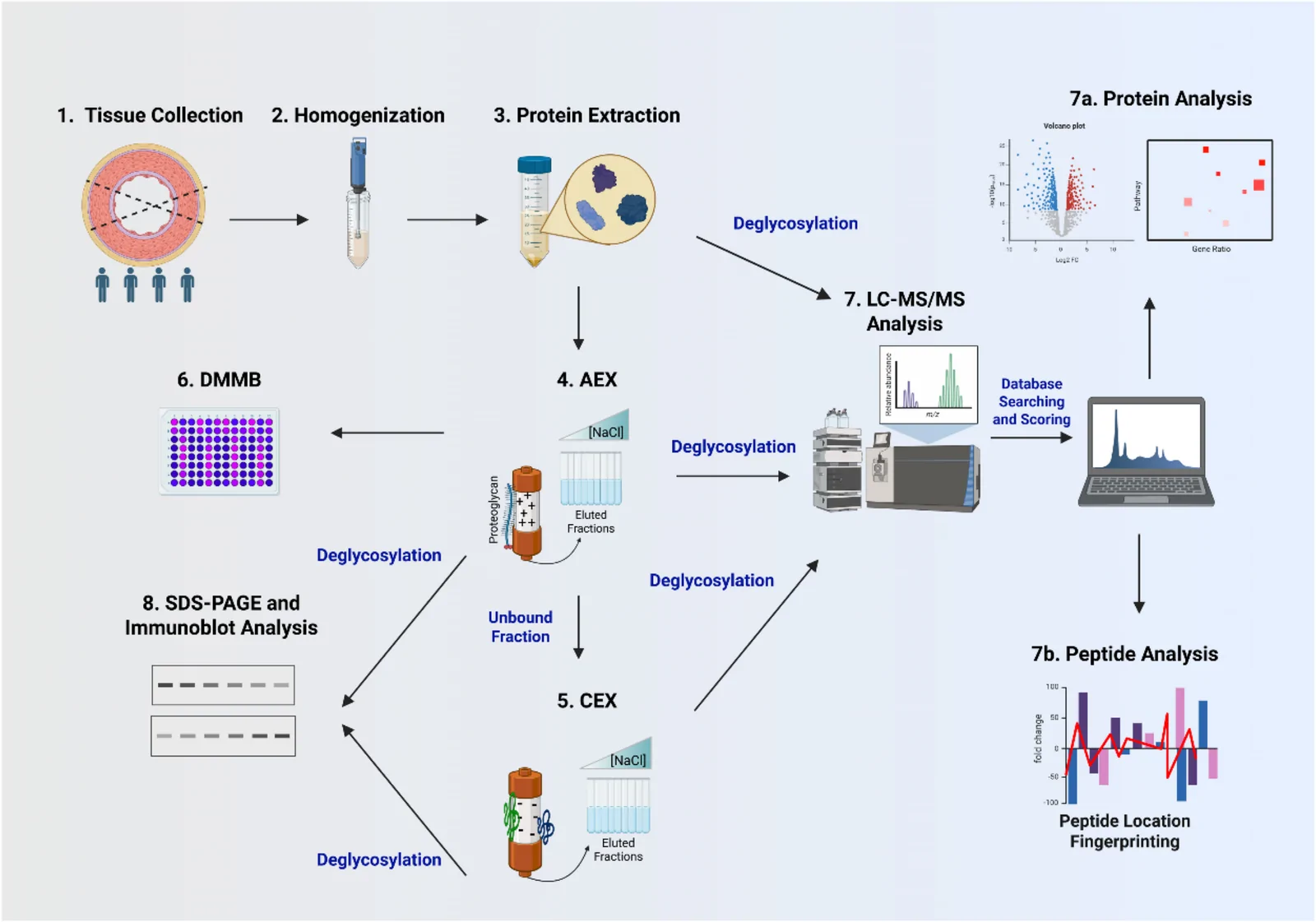
**Workflow overview.** Thoracic aortic aneurysm tissues were collected (1), homogenized (2) and subjected to protein extraction in 4 M Gu-HCl (3), followed by sequential anion (AEX, 4) and cation (CEX, 5) exchange chromatography, deglycosylation to remove glycosaminoglycans, and LC-MS/MS analysis (7). Data analysis was carried out both at protein (7a) and peptide (7b) level, the latter including Peptide Location Fingerprinting. The presence of sulfated glycosaminoglycans in AEX fractions was assessed by the dimethylmethylene blue (DMMB) assay (6), while the presence/enrichment of proteins of interest was orthogonally validated by SDS-PAGE and immunoblot assays (8). Figure created in BioRender. (For interpretation of the references to colour in this figure legend, the reader is referred to the web version of this article.)

### AEX enriches for proteoglycans

Following mechanical homogenization, proteins were extracted from TAA tissues (Supplementary Table 1) using Gu-HCl in the presence of detergents and subjected to AEX using diethylaminoethyl (DEAE) Sepharose to isolate proteoglycans. The elution profile presented two absorbance peaks at 280 nm (A_280_, Fig. 2A), hereafter indicated as ‘early’ (250–600 mM NaCl) and ‘late’ (650–900 mM NaCl) peaks. Late peaks had a consistently lower A_280_ and coincided with a single peak at 525 nm in the dimethyl methylene blue (DMMB) assay, indicating a high content of sulfated GAGs. This was confirmed by immunoblot with antibody 2B6, which recognizes the CS GAG “stubs” exposed after chondroitinase ABC treatment. Fractions eluted at higher NaCl concentrations showed increased 2B6 band intensity at molecular weights (MW) ranging from 37 to 45 kDa (Fig. 2B), indicating the presence of SLRPs and/or proteoglycan fragments.

**Fig. 2:**
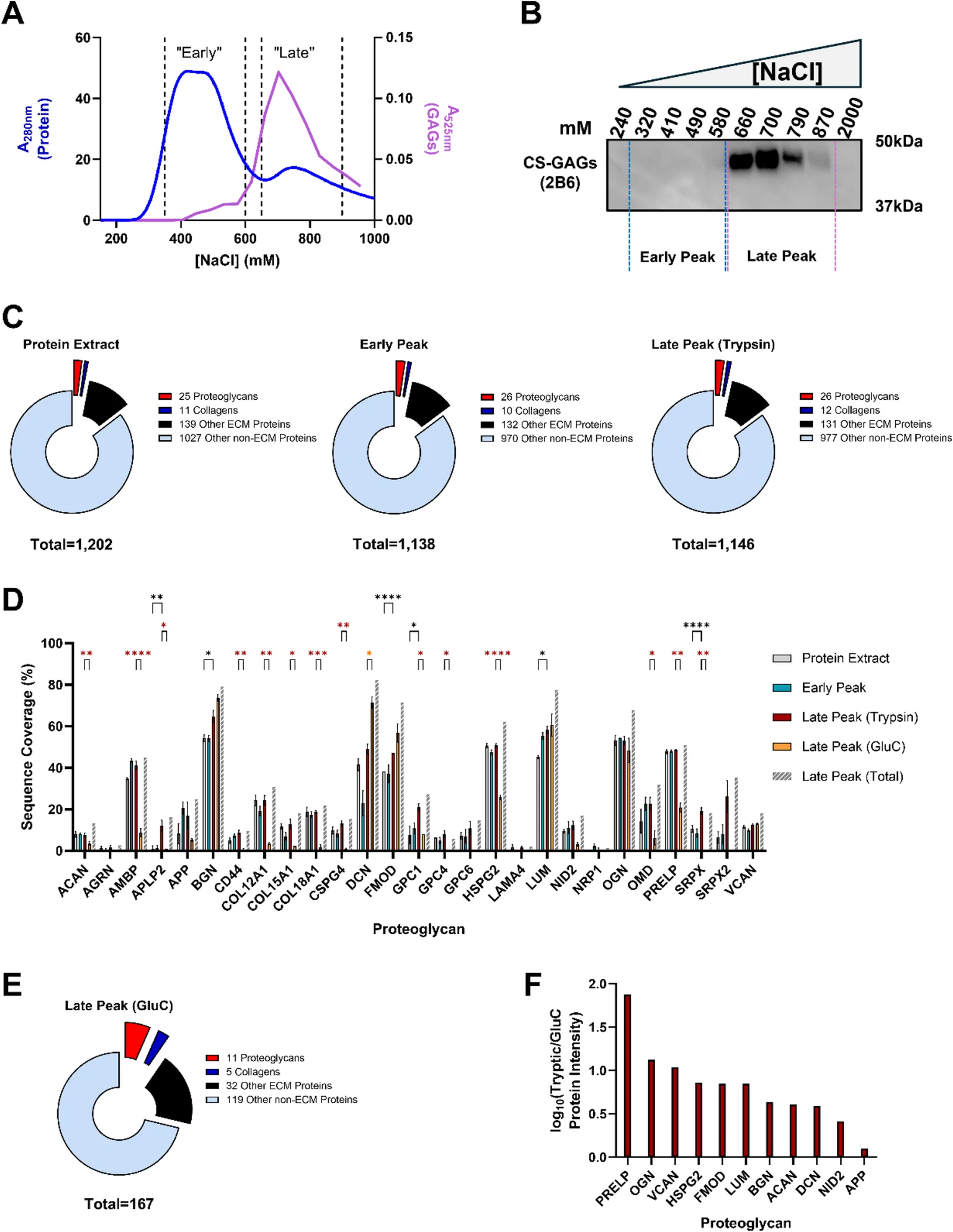
**AEX chromatography enriches in proteoglycan content. A)** Representative DEAE Sepharose absorbance at 280 nm (A_280_) chromatogram, overlayed with A_525_ values of fractions sampled at given NaCl concentrations. GAGs, glycosaminoglycans. **B)** Anti-CS-GAG (2B6) immunoblot of early and late peak fractions, showing an increase in signal with increasing NaCl concentrations. **C)** Donut plots showing the proportions and absolute numbers of total proteins, proteoglycans, collagens, other ECM proteins and non-ECM proteins identified in each fraction. **D)** Sequence coverages for proteoglycans identified across all fractions/conditions, with significant increases determined by a two-way Tukey's multiple comparison ANOVA. Late Peak (total), here shown as a reference, indicates total protein sequence computed from combined GluC/Trypsin digestions. Black asterisks: significant increases in AEX late peak vs protein extracts; dark red asterisks: significant increases in trypsin-digested vs GluC-digested AEX late peaks; orange asterisks: significant increases in GluC-digested vs trypsin-digested AEX late peaks. *: *p* < 0.05, **: *p <* 0.01, ***: *p <* 0.001, ****: *p <* 0.0001. **E)** Donut plot showing the numbers of different classes of proteins in the GluC-digested AEX late peaks. **F)** Fold changes in protein intensities between trypsin and GluC digests for proteoglycans in AEX late peaks. (For interpretation of the references to colour in this figure legend, the reader is referred to the web version of this article.)

We then compared the proteome of AEX early and late peaks with that of the protein extracts. Samples were deglycosylated prior to LC-MS/MS to improve proteoglycan coverage as previously [26]. Principal component analysis (PCA) showed clear discrimination between the 3 fractions (Supplementary Fig. 1). In total, 1305 proteins were identified: 1202 in the protein extract, 1138 in the AEX early peak and 1146 in the AEX late peak. Of these, 173, 164, and 166, respectively, were defined as matrisome proteins, including proteoglycans and collagens (Fig. 2C). Comparison of protein identifications between fractions demonstrated considerable overlap (Supplementary Fig. 2). Twenty-seven proteoglycans, including 4 transmembrane proteoglycans not reported in the MatrisomeDB [17] (APP, APLP2, NRP1, and CD44), were identified across the 3 fractions, with 26 of them identified in the AEX late and early peaks and 25 in the protein extracts (including proteins classified as both proteoglycans and collagens: COL12A1, COL15A1 and COL18A1). Compared to protein extracts, the AEX late peak showed significantly improved protein sequence coverage for 6 proteoglycans: APLP2, BGN (biglycan), FMOD (fibromodulin), GPC1, LUM (lumican), and SRPX (Fig. 2D).

As the hyalectans aggrecan and versican have a low abundance of tryptic cleavage sites (arginine and lysine residues) and high abundance of GluC sites (aspartate and glutamate residues) compared to the rest of the human proteome [11], we tested if digestion with GluC instead of trypsin increased proteoglycan sequence coverage in the AEX late peak samples. No significant differences were observed for aggrecan and versican (Fig. 2D), as previously observed in GluC digestions of purified proteoglycans [11]. On the other hand, trypsin identified 979 more proteins in the AEX late peaks, including 15 more proteoglycans and 119 more total matrisome proteins than corresponding GluC samples (Fig. 2D and E). The sequence coverages of 14 identified proteoglycans were significantly higher in the tryptic digests compared to GluC. GluC digestion significantly increased total sequence coverage only for the SLRP DCN (decorin) by 22.5% (Fig. 2D). Of the 11 proteoglycans identified following GluC digestion, all had lower (1.3–75-fold) total protein intensity compared to the tryptic digests (Fig. 2F). Based on these results, trypsin was chosen as the digestion protease for subsequent analysis.

When ranked by overall abundance, proteoglycans accounted for 36 and 27% of the cumulative abundance of the 10 and 50 most abundant proteins of the AEX late peaks compared to 6.9 and 9.8%, respectively, in protein extracts. Proteoglycans also accounted for 24% of the total proteome abundance in AEX late peaks, compared to 8.6% in protein extracts (Fig. 3A-F). Compared to protein extracts, AEX late peaks had a significantly increased abundance in the SLRPs lumican, decorin, and biglycan, and a significantly decreased abundance in 4 collagens (COL4A2, COL6A1, COL6A2, COL6A3, Fig. 3G and Table 1). There was a significantly increased abundance in biglycan and decorin in the AEX late peaks compared to the AEX early peaks, while lumican and versican abundances were similar (Fig. 3H and Table 1).

**Fig. 3:**
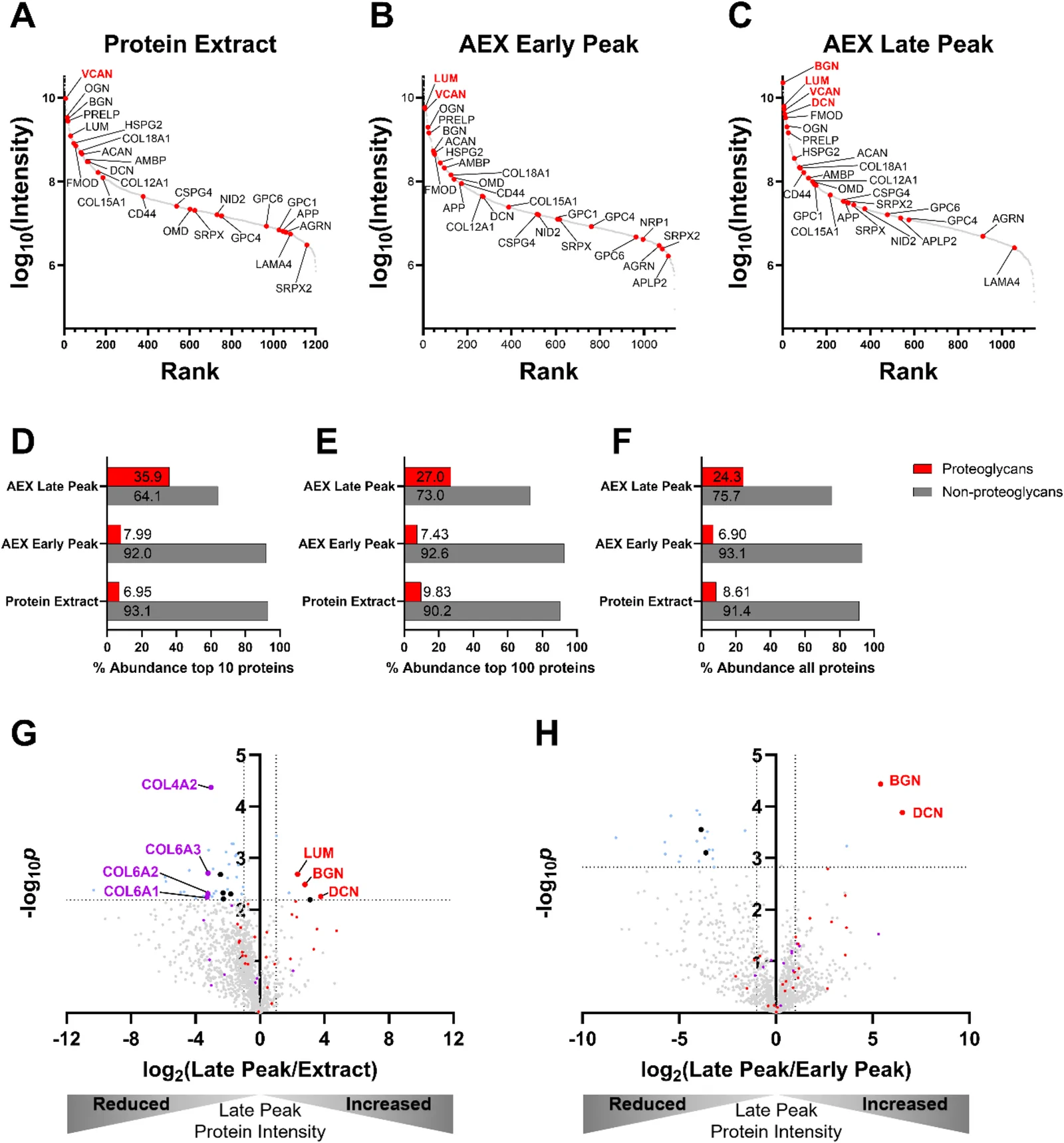
**AEX late peaks are enriched in specific proteoglycans and depleted in collagens. A-C)** Protein intensity ranks for each fraction, with proteoglycans shown as red dots, and those in the top 10 most abundant proteins labelled in red. **D-F)** Cumulative abundance (%) of proteoglycans and non-proteoglycans in the top 10 (D) and 100 (E) most abundant proteins as well as in the total proteome (F) for each fraction. **G-H)** volcano plots showing differences between protein extracts and AEX late peak (G) and between AEX late and early peaks (H). Grey points: non-significantly changed proteins; red points: proteoglycans, large red points: significantly increased/decreased proteoglycans; purple points: collagens; large purple points: significantly increased/decreased collagens, black points: significantly increased/decreased matrisome proteins; pale blue proteins: non-ECM significantly increased/decreased proteins. Dotted lines indicate significance thresholds. (For interpretation of the references to colour in this figure legend, the reader is referred to the web version of this article.)

**Table 1:** **Significant changes in protein intensities of proteoglycans and collagens across protein extracts, AEX early and late peak fractions.** Significant *p*-values, as determined by permutation-based FDR, are shown in bold.

				AEX late peak vs protein extract		AEX late peak vs early peak	
Gene Name	Protein Name	UniProt ID	Matrisome Class	log_2_ Fold Change	Direction of change (*p*-value)	log_2_ Fold Change	Direction of change (*p-*value)
*BGN*	Biglycan	P21810	proteoglycan	2.79	**increase (3.24 × 10**^**−3**^**)**	5.42	**increase (3.63 × 10**^**−5**^**)**
*DCN*	Decorin	P07585	proteoglycan	3.78	**increase (5.56 × 10**^**−3**^**)**	6.54	**increase (1.31 × 10**^**−4**^**)**
*LUM*	Lumican	P51884	proteoglycan	2.34	**increase (2.04 × 10**^**−3**^**)**	0.09	none (7.81 × 10^−1^)
*COL4A2*	Collagen alpha-2(IV) chain	P08572	collagen	−3.03	**decrease (4.25 × 10**^**−5**^**)**	−0.65	none (1.29 × 10^−1^)
*COL6A1*	Collagen alpha-1(VI) chain	P12109	collagen	−3.27	**decrease (5.72 × 10**^**−3**^**)**	0.83	none (6.90 × 10^−2^)
*COL6A2*	Collagen alpha-2(VI) chain	P12110	collagen	−3.23	**decrease (4.85 × 10**^**−3**^**)**	1.09	none (4.54 × 10^−2^)
*COL6A3*	Collagen alpha-3(VI) chain	P12111	collagen	−3.22	**decrease (1.94 × 10**^**−3**^**)**	0.41	none (1.08 × 10^−1^)

Changes in proteoglycan abundances across the 3 fractions were orthogonally supported by immunoblot analysis, with increased band intensity observed for CS proteoglycans decorin and biglycan in the AEX late peaks compared with AEX early peaks and protein extracts (Fig. 4A). Detection of high MW (i.e., >150 kDa) versican species was also increased in the AEX late peaks compared with protein extracts and AEX early peaks (Fig. 4B). Immunoblotting for versikine, a versican fragment generated by a disintegrin-like and metalloprotease with thrombospondin motifs (ADAMTS)-mediated proteolysis, and lacking CS-GAGs [27], revealed an increased abundance in protein extracts and AEX early peaks compared with the AEX late peaks. Based on molecular weight, V1-derived versikine (~70 kDa) was the predominant form detected across all samples, as the expected size of V0-derived versikine is around 220 kDa [27] (Fig. 4B). For aggrecan, the intensity of low MW (i.e., <100 kDa) bands decreased in the AEX late peaks compared to the protein extracts, while intensity of high MW bands was comparable (Fig. 4C).

**Fig. 4:**
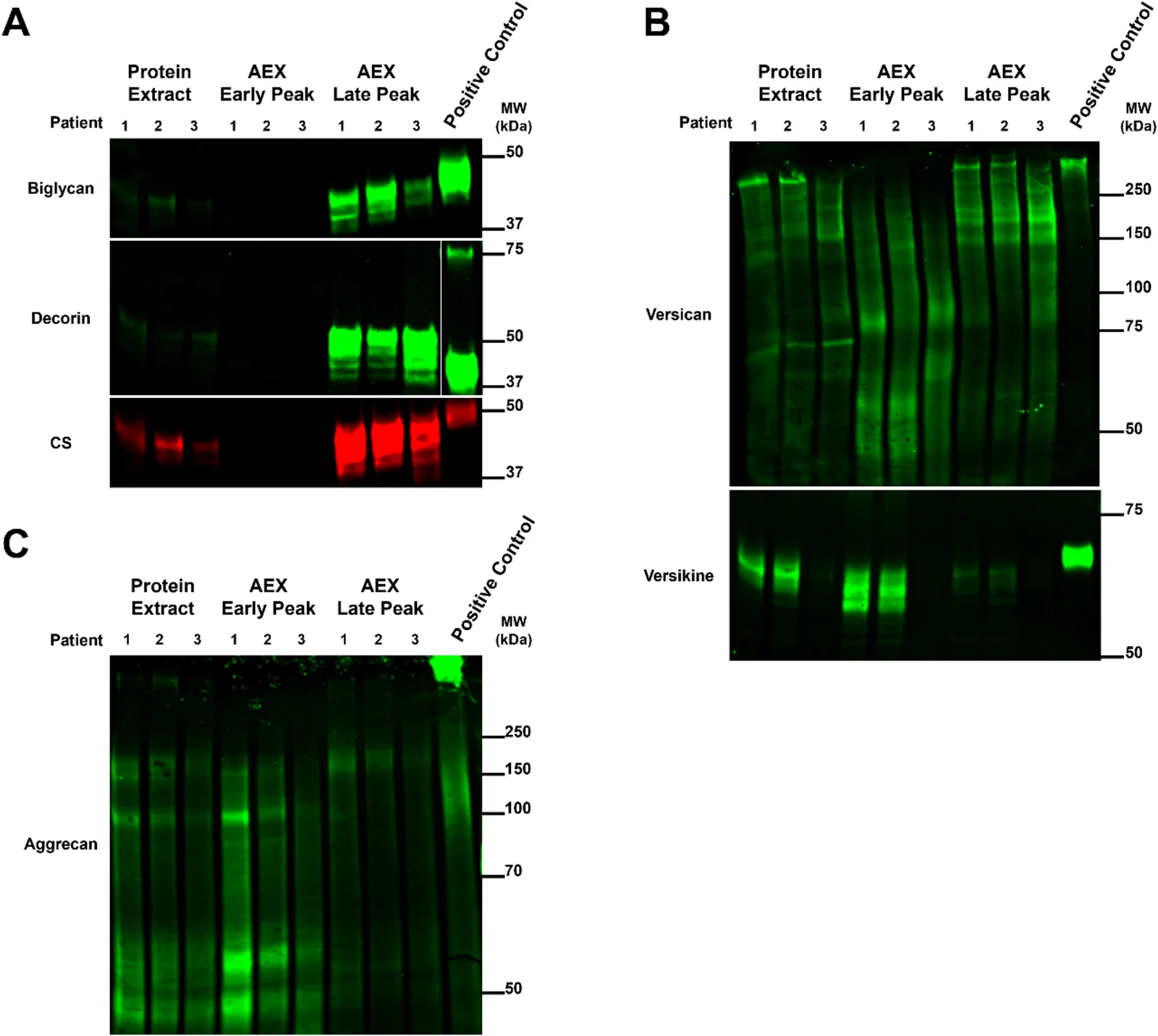
**Orthogonal validation of proteoglycan enrichment in AEX.** A) Anti-biglycan, anti-decorin, anti-CS/2B6, B) anti-versican, anti-versikine and C) anti-aggrecan immunoblots of protein extracts and AEX peaks. Numbers above lanes indicate individual patients. MW, molecular weight.

Sequence coverage of large aggregating proteoglycans and SLRPs was assessed by PLF [28]. This analysis revealed significantly higher peptide intensities in the AEX late peaks relative to the protein extracts across biglycan (7/15 non-zero bins, Fig. 5A), lumican (8/15 non-zero bins, Fig. 5B) and decorin (6/12 non-zero bins, Fig. 5E), consistent with their increased protein abundance (Fig. 3G and Fig. 4A). Peptide intensities fluctuated along versican protein sequence, with higher intensity in the AEX late peaks in the C-terminal G3 domain, possibly suggesting the presence of full-length species, though these did not reach statistical significance. Significantly higher bin intensity was observed for the versican N-terminal G1 domain in the protein extracts (Fig. 5C**)**. In the AEX late peaks, aggrecan showed a significantly lower intensity in the N-terminal G1 domains with little coverage in its glycosylated CS1 and CS2 domains, but higher intensity in the C-terminal G3 domain compared with the protein extracts (Fig. 5D**)**, suggesting enrichment for C-terminal fragments and/or full-length aggrecan.

**Fig. 5:**
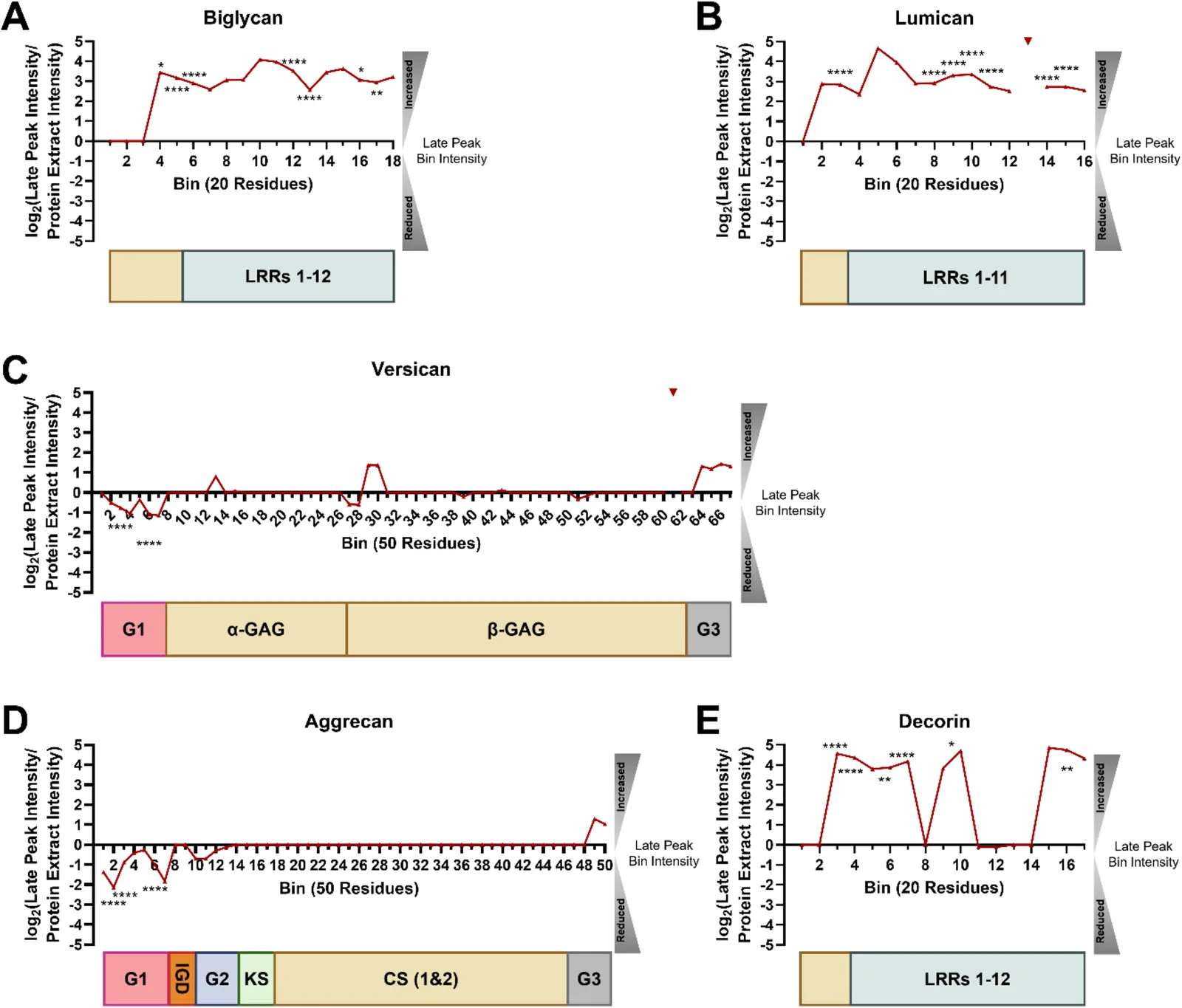
**Peptide Location Fingerprinting analysis of TAA-relevant proteoglycans. A)** biglycan (20 residues/bin), **B)** lumican (20 residues/bin) **C)** versican (50 residues/bin), **D)** aggrecan (50 residues/bin) and **E)** decorin (20 residues/bin), with their schematic domain organization overlayed underneath. Increases in AEX late peak (red line, top graphs A-E) intensity, per bin, are shown above the *x*-axis, increases in protein extract intensities are shown below. Values are reported as log_2_(fold-changes) for visualization purposes. Bins which are present in the late peaks and absent in the protein extracts are indicated by an inverted triangle. GAG, glycosaminoglycan; CS, chondroitin sulfate; KS, keratan sulfate; LRR, leucine-rich repeat; IGD, interglobular domain. Significance was determined by a two-way repeated measures ANOVA per protein, on mean overall bin intensities, with Bonferroni-corrected multiple comparisons. *: *p* < 0.05, **: *p* < 0.01, ****: *p* < 0.0001. (For interpretation of the references to colour in this figure legend, the reader is referred to the web version of this article.)

Mean intensity values per bin for each condition are shown in Supplementary Figs. 3–7.

Based on the epitope information provided by the different antibody manufacturers, we were able to partially support our PLF analysis with immunoblot data. The significantly higher decorin peptide intensities observed in AEX late peaks across bins 3–4 (residues 41–80) and 6–7 (residues 101–140) (Fig. 5E) are consistent with immunoblot data (Fig. 4A), as the epitope recognized by the decorin antibody (residues 17–172) overlaps substantially with this region. The biglycan antibody epitope (residues 350–368) also corresponds to an increased region in AEX late peak (bin 18) (Fig. 5A), consistent with immunoblot data, though this bin intensity increase did not reach significance. Finally, the decreased signal in the N-terminal versican G1 domain in AEX late peaks compared to protein extracts (Fig. 5C) was supported by versikine immunoblot data (Fig. 4B), as G1 makes up approximately 79% of the V1-derived versikine sequence.

Together, these results indicate that, although the diversity of the proteoglycanome is maintained in both the protein extracts and AEX fractions, AEX is effective in isolating a number of proteoglycans, with higher salt concentrations selectively enriching in specific full-length and/or high MW proteoglycan species, while depleting in collagens.

### CEX chromatography enriches for ECM Regulators, including proteases and protease inhibitors, and secreted factors

To improve detection and coverage of matrisome-associated proteins, the unbound fraction from AEX chromatography was subjected to CEX chromatography using heparin Sepharose (Fig. 1), as heparin is known to bind clusters of basic residues in secreted factors, proteases and protease inhibitors [29], [30]. Heparin-bound proteins, representing the aortic “heparin ligandome”, consistently eluted as a single peak at 750–1500 mM NaCl (Supplementary Fig. 8). CEX peaks were then subjected to deglycosylation (to allow for a direct comparison with AEX peaks), trypsinization and LC-MS/MS analysis. PCA revealed distinct separation among protein extracts, AEX late peaks and CEX peaks (Supplementary Fig. 9). In total, 1203 proteins were identified in CEX peaks, of which 187 were matrisome proteins, including 53 ECM regulators and 23 secreted factors (Fig. 6A and Supplementary Fig. 10). Across all fractions, 16 matrisome proteins were exclusively found in CEX peaks**,** including 3 ECM regulators and 7 secreted factors (5.2% and 25.9% of the total proteins identified from their protein class, respectively, Table 2, Table 3 and Supplementary Fig. 10). The proportion of secreted factors in the proteome was higher in the CEX peaks (1.9%) compared to the AEX late peaks (1.1%) and the protein extracts (1.3%). Ranking proteins by overall abundance revealed that the CEX peaks had 8 ECM regulators and 1 secreted factor within the top 100 most abundant proteins, whereas both the protein extracts and AEX late peaks had only 4 ECM regulators, and 1 and 0 secreted factors, respectively. In the top 200 most abundant proteins, the CEX peak had 7 secreted factors, compared to 2 in both the protein extracts and AEX late peaks (Fig. 6B-D). The cumulative abundance of both ECM regulators and secreted factors was increased in the CEX peaks compared to both protein extracts and AEX peaks (Fig. 6E-G). All of the proteins identified in the CEX peaks, including sequence coverages, intensities, spectral counts and unique peptides, are reported in Supplementary file 1.

**Fig. 6:**
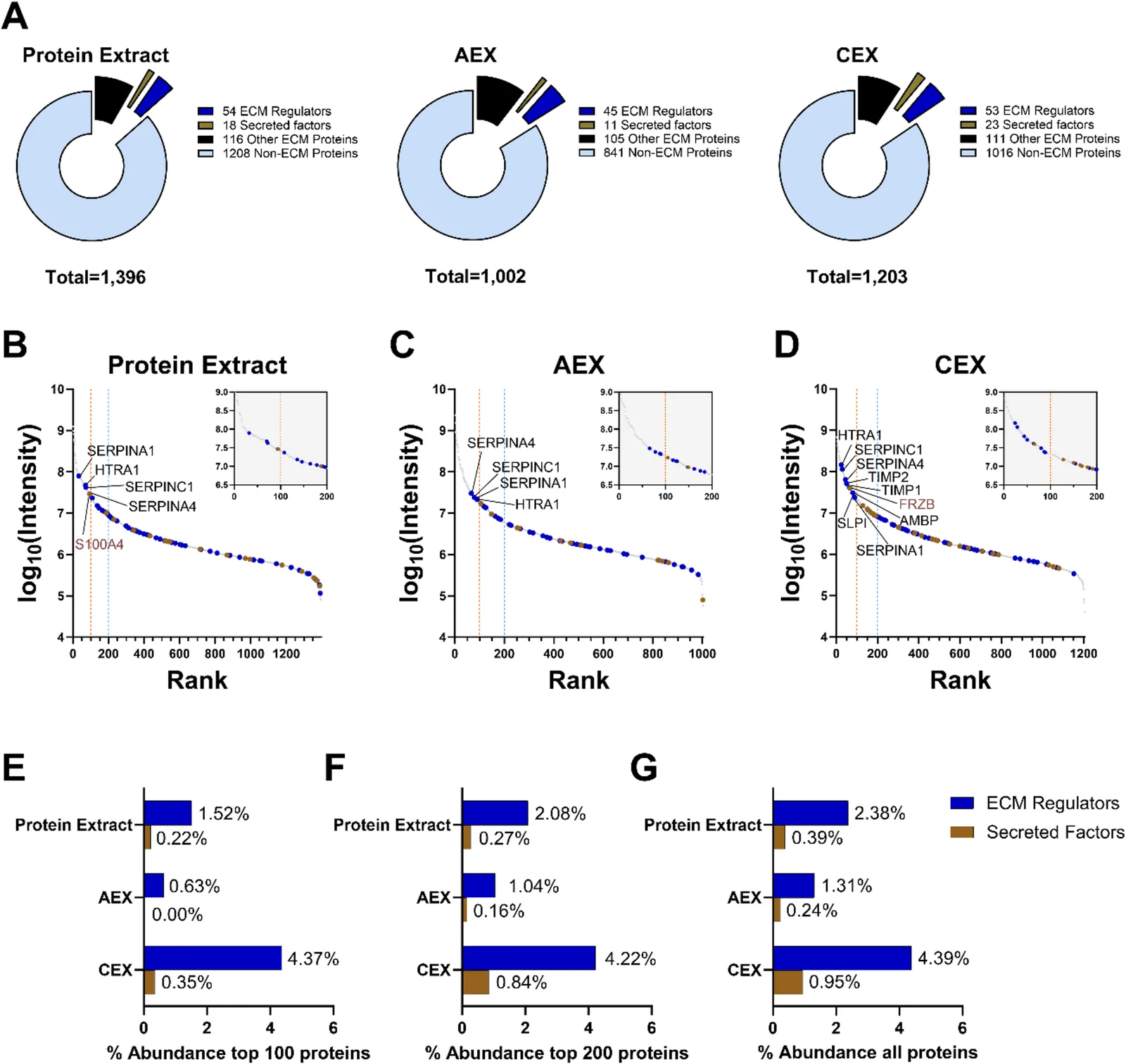
**CEX chromatography enriches in ECM regulators. A)** Donut plots of protein extracts, AEX and CEX peaks showing the composition of the proteome, including secreted factors, ECM regulators and other ECM proteins. **B–D)** rank diagrams of protein intensities across the different fractions. ECM regulators are highlighted in blue, secreted factors are highlighted in gold, and those in the top 100 identified proteins by intensity are labelled. The top 100 and 200 proteins are shown to the left of the orange and blue dashed lines, respectively. The inserts show magnification of the rank for the top 200 protein to show distribution of ECM regulators and secreted factors. **E–G)** Cumulative abundance of all ECM regulators and secreted factors in **E**) the top 100, **F**) the top 200 and **G**) all proteins identified in protein extract, AEX and CEX fractions**.** AEX, anion exchange chromatography; CEX, cation exchange chromatography. (For interpretation of the references to colour in this figure legend, the reader is referred to the web version of this article.)

**Table 2:** ECM regulators identified in protein extracts, AEX late peaks and CEX peaks.

Gene name	Protein name	UniProt ID	Presence in condition		
			Protein extract	AEX	CEX
*A2M*	Alpha-2-macroglobulin	P01023	✓	✓	✓
*ADAMTSL1*	ADAMTS-like protein 1	Q8N6G6	×	✓	✓
*ADAMTSL3*	ADAMTS-like protein 3	P82987	✓	×	✓
*ADAMTSL4*	ADAMTS-like protein 4	Q6UY14	×	×	✓
*AGT*	Angiotensinogen	P01019	✓	✓	✓
*AMBP*	Protein AMBP	P02760	✓	✓	✓
*CD109*	CD109 antigen	Q6YHK3	✓	✓	✓
*CST3*	Cystatin-C	P01034	✓	×	×
*CSTB*	Cystatin-B	P04080	✓	✓	✓
*CTSB*	Cathepsin B	P07858	✓	✓	✓
*CTSD*	Cathepsin D	P07339	✓	✓	✓
*CTSF*	Cathepsin F	Q9UBX1	✓	✓	✓
*CTSG*	Cathepsin G	P08311	✓	×	✓
*CTSZ*	Cathepsin Z	Q9UBR2	✓	✓	✓
*ELANE*	Neutrophil elastase	P08246	✓	×	✓
*F2*	Prothrombin	P00734	✓	✓	✓
*F9*	Coagulation factor IX	P00740	✓	✓	✓
*F12*	Coagulation factor XII	P00748	✓	×	✓
*F13A1*	Coagulation factor XIII A chain	P00488	✓	✓	✓
*HABP2*	Factor VII-activating protease	Q14520	✓	✓	×
*HRG*	Histidine-rich glycoprotein	P04196	✓	✓	✓
*HTRA1*	Serine protease HTRA1	Q92743	✓	✓	✓
*ITIH1*	Inter-alpha-trypsin inhibitor heavy chain H1	P19827	✓	✓	✓
*ITIH2*	Inter-alpha-trypsin inhibitor heavy chain H2	P19823	✓	✓	✓
*ITIH3*	Inter-alpha-trypsin inhibitor heavy chain H3	Q06033	✓	✓	✓
*ITIH4*	Inter-alpha-trypsin inhibitor heavy chain H4	Q14624	✓	✓	✓
*ITIH5*	Inter-alpha-trypsin inhibitor heavy chain H5	Q86UX2	✓	✓	✓
*KNG1*	Kininogen-1	P01042	✓	✓	✓
*LOXL1*	Lysyl oxidase homolog 1	Q08397	✓	✓	✓
*MASP1*	Mannan-binding lectin serine protease 1	P48740	✓	✓	✓
*MMP2*	72 kDa type IV collagenase	P08253	✓	✓	✓
*PAPPA*	Pappalysin-1	Q13219	×	×	✓
*PLG*	Plasminogen	P00747	✓	✓	✓
*PLOD1*	Procollagen-lysine,2-oxoglutarate 5-dioxygenase 1	Q02809	✓	✓	×
*SERPINA1*	Alpha-1-antitrypsin	P01009	✓	✓	✓
*SERPINA3*	Alpha-1-antichymotrypsin	P01011	✓	✓	✓
*SERPINA4*	Kallistatin	P29622	✓	✓	✓
*SERPINA5*	Plasma serine protease inhibitor	P05154	✓	✓	✓
*SERPINA6*	Corticosteroid-binding globulin	P08185	✓	✓	✓
*SERPINA7*	Thyroxine-binding globulin	P05543	✓	✓	×
*SERPINA10*	Protein *Z*-dependent protease inhibitor	Q9UK55	✓	×	✓
*SERPINB1*	Leukocyte elastase inhibitor	P30740	✓	✓	✓
*SERPINB6*	Serpin B6	P35237	✓	✓	✓
*SERPINB9*	Serpin B9	P50453	✓	×	×
*SERPINC1*	Antithrombin-III	P01008	✓	✓	✓
*SERPIND1*	Heparin cofactor 2	P05546	✓	✓	✓
*SERPINE1*	Plasminogen activator inhibitor 1	P05121	✓	×	✓
*SERPINE2*	Glia-derived nexin	P07093	✓	✓	✓
*SERPINF1*	Pigment epithelium-derived factor	P36955	✓	✓	✓
*SERPINF2*	Alpha-2-antiplasmin	P08697	✓	✓	✓
*SERPING1*	Plasma protease C1 inhibitor	P05155	✓	✓	✓
*SERPINH1*	Serpin H1	P50454	✓	✓	✓
*SLPI*	Antileukoproteinase	P03973	✓	✓	✓
*SULF1*	Extracellular sulfatase Sulf-1	Q8IWU6	×	×	✓
*TGM2*	Protein-glutamine gamma-glutamyltransferase 2	P21980	✓	✓	✓
*TIMP1*	Metalloproteinase inhibitor 1	P01033	✓	×	✓
*TIMP2*	Metalloproteinase inhibitor 2	P16035	✓	×	✓
*TIMP3*	Metalloproteinase inhibitor 3	P35625	✓	✓	✓

**Table 3:** ECM secreted factors identified in protein extracts, AEX peaks and CEX peaks.

			Presence in condition		
Gene name	Protein name	UniProt ID	Protein extract	AEX	CEX
*ANGPTL4*	Angiopoietin-related protein 4	Q9BY76	×	×	✓
*CCL14*	C-C motif chemokine 14	Q16627	✓	×	✓
*CCL16*	C-C motif chemokine 16	O15467	×	×	✓
*CCL18*	C-C motif chemokine 18	P55774	✓	×	✓
*CXCL12*	Stromal cell-derived factor 1	P48061	✓	✓	✓
*FGF1*	Fibroblast growth factor 1	P05230	✓	×	×
*FRZB*	Secreted frizzled-related protein 3	Q92765	✓	✓	✓
*FSTL1*	Follistatin-related protein 1	Q12841	✓	×	✓
*HCFC1*	Host cell factor 1	P51610	✓	×	×
*HGF*	Hepatocyte growth factor	P14210	×	×	✓
*IGF2*	Insulin-like growth factor 2	P01344	×	×	✓
*IL34*	Interleukin-34	Q6ZMJ4	✓	✓	×
*MEGF6*	Multiple epidermal growth factor-like domains protein 6	O75095	✓	✓	✓
*MST1*	Hepatocyte growth factor-like protein	P26927	✓	✓	✓
*PPBP*	Platelet basic protein	P02775	✓	×	✓
*S100A4*	Protein S100-A4	P26447	✓	✓	✓
*S100A6*	Protein S100-A6	P06703	✓	×	✓
*S100A9*	Protein S100-A9	P06702	×	✓	✓
*S100A10*	Protein S100-A10	P60903	✓	×	✓
*S100A11*	Protein S100-A11	P31949	✓	✓	✓
*S100A13*	Protein S100-A13	Q99584	×	×	✓
*SCUBE1*	Signal peptide, CUB and EGF-like domain-containing protein 1	Q8IWY4	×	×	✓
*SCUBE3*	Signal peptide, CUB and EGF-like domain-containing protein 3	Q8IX30	✓	✓	✓
*SFRP1*	Secreted frizzled-related protein 1	Q8N474	✓	✓	✓
*SFRP2*	Secreted frizzled-related protein 2	Q96HF1	×	×	✓
*SFRP4*	Secreted frizzled-related protein 4	Q6FHJ7	✓	×	✓
*TGFB1*	Transforming growth factor beta-1 proprotein	P01137	×	✓	×

Compared to both the protein extracts and the AEX late peaks, the abundance of 10 and 8 ECM secreted factors, and 14 and 12 ECM regulators, respectively, significantly increased in CEX. These included proteases (HtrA serine peptidase 1, HTRA1, and neutrophil elastase), protease inhibitors (tissue inhibitor of metalloproteinase-1/TIMP-1 and TIMP-2), and ADAMTS-like 4 (ADAMTSL4). The secreted factors significantly enriched in the CEX peaks included C—C motif chemokine 14 (CCL14), CCL16, insulin-like growth factor 2 (IGF2), hepatocyte growth factor-like protein (MST1) and S100A13 (Table 4).

**Table 4:** **Significant changes in CEX peak matrisome protein intensities compared to AEX peaks or protein extracts.** Significant changes were determined by FDR-based permutation, significant *p-*values are shown in bold.

				CEX vs AEX late peak		CEX vs Protein Extract	
Gene Name	Protein Name	UniProt ID	Class	log_2_ Fold Change	Direction of change (*p-*value)	log_2_ Fold Change	Direction of change (*p*-value)
*ACAN*	Aggrecan core protein	P16112	Proteoglycans	-3.80	**decrease (2.53×10**^**-2**^**)**	-3.86	**decrease (3.14×10**^**-4**^**)**
*ADAMTSL3*	ADAMTS-like protein 3	P82987	**ECM Regulators**	4.51	none (7.18×10^-2^)	2.77	**increase (3.47×10**^**-2**^**)**
*ADAMTSL4*	ADAMTS-like protein 4	Q6UY14	**ECM Regulators**	4.00	**increase (8.77×10**^**-3**^**)**	3.30	**increase (1.08×10**^**-2**^**)**
*ANXA1*	Annexin A1	P04083	ECM-Affiliated Proteins	1.40	**increase (8.66×10**^**-3**^**)**	-1.62	**decrease (1.69×10**^**-2**^**)**
*ANXA4*	Annexin A4	P09525	ECM-Affiliated Proteins	0.01	none (9.71×10^-1^)	-1.73	**decrease (5.84×10**^**-3**^**)**
*ANXA5*	Annexin A5	P08758	ECM-Affiliated Proteins	-0.82	none (1.03×10^-1^)	-1.48	**decrease (1.56×10**^**-2**^**)**
*BGN*	Biglycan	P21810	Proteoglycans	-5.15	**decrease (1.28×10**^**-3**^**)**	-1.57	**decrease (1.30×10**^**-2**^**)**
*CCL14*	C-C motif chemokine 14	Q16627	**Secreted Factors**	7.14	**increase (6.13×10**^**-6**^**)**	5.42	**increase (2.28×10**^**-3**^**)**
*CCL16*	C-C motif chemokine 16	O15467	**Secreted Factors**	7.05	**increase (7.97×10**^**-3**^**)**	4.36	**increase (4.36×10**^**-4**^**)**
*CCL18*	C-C motif chemokine 18	P55774	**Secreted Factors**	4.43	none (1.75×10^-1^)	2.04	**increase (4.29×10**^**-2**^**)**
*CLEC11A*	C-type lectin domain family 11 member A	Q9Y240	ECM-Affiliated Proteins	-2.89	**decrease (1.19×10**^**-2**^**)**	-1.89	**decrease (7.99×10**^**-3**^**)**
*CLEC3B*	Tetranectin	P05452	ECM-Affiliated Proteins	4.64	**increase (3.46×10**^**-3**^**)**	-0.78	none (2.29×10^-1^)
*COL12A1*	Collagen alpha-1(XII) chain	Q99715	Collagens, Proteoglycans	1.56	**increase (3.70×10**^**-2**^**)**	2.05	**increase (4.27×10**^**-4**^**)**
*COL14A1*	Collagen alpha-1(XIV) chain	Q05707	Collagens	0.98	none (1.21×10^-2^)	1.86	**increase (1.31×10**^**-3**^**)**
*COL15A1*	Collagen alpha-1(XV) chain	P39059	Collagens, Proteoglycans	-3.20	none (7.33×10^-2^)	-2.80	**decrease (5.63×10**^**-2**^**)**
*COL16A1*	Collagen alpha-1(XVI) chain	Q07092	Collagens	4.74	**increase (3.25×10**^**-2**^**)**	2.60	**increase (8.35×10**^**-4**^**)**
*COL18A1*	Collagen alpha-1(XVIII) chain	P39060	Collagens, Proteoglycans	3.45	**increase (9.25×10**^**-4**^**)**	2.53	**increase (1.46×10**^**-2**^**)**
*COL1A1*	Collagen alpha-1(I) chain	P02452	Collagens	4.74	none (9.55×10^-2^)	1.93	**increase (1.13×10**^**-2**^**)**
*COL1A2*	Collagen alpha-2(I) chain	P08123	Collagens	3.16	**increase (1.86×10**^**-3**^**)**	0.95	none (1.98×10^-1^)
*COL21A1*	Collagen alpha-1(XXI) chain	Q96P44	Collagens	5.36	**increase (6.47×10**^**-5**^**)**	5.98	**increase (8.53×10**^**-3**^**)**
*COL3A1*	Collagen alpha-1(III) chain	P02461	Collagens	2.63	**increase (2.31×10**^**-4**^**)**	3.59	**increase (1.30×10**^**-2**^**)**
*COL4A1*	Collagen alpha-1(IV) chain	P02462	Collagens	0.18	none (6.81×10^-1^)	-2.50	**decrease (7.16×10**^**-4**^**)**
*COL4A2*	Collagen alpha-2(IV) chain	P08572	Collagens	0.70	none (1.26×10^-1^)	-1.59	**decrease (1.77×10**^**-3**^**)**
*COL5A1*	Collagen alpha-1(V) chain	P20908	Collagens	-5.08	**decrease (8.65×10**^**-4**^**)**	0.19	none (8.59×10^-1^)
*COL8A1*	Collagen alpha-1(VIII) chain	P27658	Collagens	1.50	**increase (1.05×10**^**-2**^**)**	0.42	none (3.43×10^-1^)
*CSPG4*	Chondroitin sulfate proteoglycan 4	Q6UVK1	Proteoglycans, ECM-Affiliated Proteins	-3.20	**decrease (1.05×10**^**-2**^**)**	-1.83	**decrease (5.35×10**^**-2**^**)**
*CST3*	Cystatin-C	P01034	**ECM Regulators**	0.31	none (6.28×10^-1^)	-5.44	**decrease (9.02×10**^**-4**^**)**
*CTHRC1*	Collagen triple helix repeat-containing protein 1	Q96CG8	ECM Glycoproteins	1.98	**increase (1.79×10**^**-2**^**)**	1.16	**increase (2.53×10**^**-2**^**)**
*DCN*	Decorin	P07585	Proteoglycans	-8.14	**decrease (2.94×10**^**-4**^**)**	-3.90	**decrease (3.21×10**^**-3**^**)**
*DPT*	Dermatopontin	Q07507	ECM Glycoproteins	-10.76	**decrease (1.50×10**^**-4**^**)**	-9.08	**decrease (2.61×10**^**-4**^**)**
*ECM1*	Extracellular matrix protein 1	Q16610	ECM Glycoproteins	6.09	**increase (3.48×10**^**-6**^**)**	3.91	**increase (3.04×10**^**-3**^**)**
*ELANE*	Neutrophil elastase	P08246	**ECM Regulators**	5.35	**increase (4.71×10**^**-3**^**)**	3.88	**increase (7.12×10**^**-3**^**)**
*ELN*	Elastin	P15502	ECM Glycoproteins	1.11	none (4.82×10^-1^)	-3.65	**decrease (3.69×10**^**-2**^**)**
*EMILIN1*	EMILIN-1	Q9Y6C2	ECM Glycoproteins	-3.55	**decrease (3.49×10**^**-2**^**)**	-2.47	**decrease (6.18×10**^**-2**^**)**
*F2*	Prothrombin	P00734	**ECM Regulators**	-0.60	none (1.18×10^-1^)	-1.89	**decrease (4.81×10**^**-4**^**)**
*F9*	Coagulation factor IX	P00740	**ECM Regulators**	-0.92	none (5.49×10^-2^)	-1.71	**decrease (2.10×10**^**-3**^**)**
*FBLN1*	Fibulin-1	P23142	ECM Glycoproteins	-2.02	**decrease (2.37×10**^**-3**^**)**	-0.93	none (1.41×10^-2^)
*FBLN5*	Fibulin-5	Q9UBX5	ECM Glycoproteins	-1.69	**decrease (6.92×10**^**-3**^**)**	-0.84	none (5.52×10^-5^)
*FBN1*	Fibrillin-1	P35555	ECM Glycoproteins	-3.40	**decrease (2.54×10**^**-3**^**)**	-3.06	**decrease (3.28×10**^**-3**^**)**
*FMOD*	Fibromodulin	Q06828	Proteoglycans	-5.13	**decrease (4.57×10**^**-4**^**)**	-1.81	**decrease (4.44×10**^**-3**^**)**
*FN1*	Fibronectin	P02751	ECM Glycoproteins	0.57	none (2.41×10^-1^)	1.38	**increase (8.37×10**^**-3**^**)**
*FNDC1*	Fibronectin type III domain-containing protein 1	Q4ZHG4	ECM Glycoproteins	-2.59	**decrease (2.78×10**^**-2**^**)**	2.11	none (2.70×10^-1^)
*FSTL1*	Follistatin-related protein 1	Q12841	**Secreted Factors**	3.56	**increase (3.74×10**^**-2**^**)**	-0.02	none (9.73×10^-1^)
*GPC1*	Glypican-1	P35052	Proteoglycans, ECM-Affiliated Proteins	-5.29	**decrease (7.79×10**^**-5**^**)**	-2.76	**decrease (7.12×10**^**-3**^**)**
*HABP2*	Factor VII-activating protease	Q14520	**ECM Regulators**	-5.46	**decrease (3.46×10**^**-4**^**)**	-1.29	**decrease (4.35×10**^**-3**^**)**
*HCFC1*	Host cell factor 1	P51610	**Secreted Factors**	-0.71	none (4.39×10^-1^)	-1.95	**decrease (7.27×10**^**-2**^**)**
*HGF*	Hepatocyte growth factor	P14210	**Secreted Factors**	3.15	none (5.59×10^-2^)	2.69	**increase (5.91×10**^**-3**^**)**
*HMCN1*	Hemicentin-1	Q96RW7	ECM Glycoproteins	2.33	**increase (3.16×10**^**-2**^**)**	0.28	none (6.59×10^-1^)
*HPX*	Hemopexin	P02790	ECM-Affiliated Proteins	0.51	none (2.53×10^-1^)	-2.04	**decrease (1.77×10**^**-3**^**)**
*HRG*	Histidine-rich glycoprotein	P04196	**ECM Regulators**	2.69	**increase (3.53×10**^**-2**^**)**	0.02	none (9.83×10^-1^)
*HSPG2*	Basement membrane-specific heparan sulfate proteoglycan core protein	P98160	Proteoglycans	-1.79	**decrease (1.20×10**^**-2**^**)**	-1.75	**decrease (7.13×10**^**-3**^**)**
*HTRA1*	Serine protease HTRA1	Q92743	**ECM Regulators**	2.77	**increase (2.94×10**^**-3**^**)**	1.63	**increase (4.54×10**^**-2**^**)**
*IGF2*	Insulin-like growth factor 2	P01344	**Secreted Factors**	5.09	**increase (7.37×10**^**-5**^**)**	5.91	**increase (1.48×10**^**-3**^**)**
*IGFBP2*	Insulin-like growth factor-binding protein 2	P18065	ECM Glycoproteins	5.24	**increase (2.84×10**^**-3**^**)**	1.67	**increase (2.62×10**^**-2**^**)**
*IGFBP3*	Insulin-like growth factor-binding protein 3	P17936	ECM Glycoproteins	4.33	**increase (4.69×10**^**-3**^**)**	3.55	**increase (7.19×10**^**-3**^**)**
*IGFBP6*	Insulin-like growth factor-binding protein 6	P24592	ECM Glycoproteins	7.96	**increase (1.96×10**^**-5**^**)**	3.66	**increase (2.17×10**^**-3**^**)**
*IGFBP7*	Insulin-like growth factor-binding protein 7	Q16270	ECM Glycoproteins	7.25	**increase (1.83×10**^**-4**^**)**	3.74	**increase (3.95×10**^**-3**^**)**
*IL34*	Interleukin-34	Q6ZMJ4	**Secreted Factors**	-3.52	**decrease (2.35×10**^**-2**^**)**	-2.04	none (8.69×10^-2^)
*KNG1*	Kininogen-1	P01042	**ECM Regulators**	0.13	none (8.26×10^-1^)	-2.34	**decrease (3.09×10**^**-3**^**)**
*LGALS1*	Galectin-1	P09382	ECM-Affiliated Proteins	0.23	none (2.39×10^-1^)	-1.62	**decrease (1.44×10**^**-3**^**)**
*LGALS3*	Galectin-3	P17931	ECM-Affiliated Proteins	2.34	**increase (3.17×10**^**-2**^**)**	-1.00	none (2.00×10^-1^)
*LOXL1*	Lysyl oxidase homolog 1	Q08397	**ECM Regulators**	1.74	**increase (3.68×10**^**-2**^**)**	-1.43	**decrease (2.04×10**^**-2**^**)**
*LTBP1*	Latent-transforming growth factor beta-binding protein 1	Q14766	ECM Glycoproteins	-1.27	**decrease (3.80×10**^**-3**^**)**	-0.70	none (6.91×10^-2^)
*LTBP4*	Latent-transforming growth factor beta-binding protein 4	Q8N2S1	ECM Glycoproteins	-3.87	**decrease (7.24×10**^**-5**^**)**	-3.14	**decrease (1.54×10**^**-3**^**)**
*LUM*	Lumican	P51884	Proteoglycans	-5.42	**decrease (7.25×10**^**-5**^**)**	-2.36	**decrease (3.62×10**^**-3**^**)**
*MATN2*	Matrilin-2	O00339	ECM Glycoproteins	0.49	none (3.01×10^-1^)	1.22	**increase (4.54×10**^**-2**^**)**
*MST1*	Hepatocyte growth factor-like protein	P26927	**Secreted Factors**	5.99	**increase (1.15×10**^**-2**^**)**	3.68	**increase (2.59×10**^**-2**^**)**
*NID2*	Nidogen-2	Q14112	Proteoglycans, ECM Glycoproteins	-1.49	**decrease (2.18×10**^**-3**^**)**	-0.68	none (2.46×10^-2^)
*NPNT*	Nephronectin	Q6UXI9	ECM Glycoproteins	1.17	**increase (2.09×10**^**-2**^**)**	1.35	**increase (3.05×10**^**-2**^**)**
*PAPPA*	Pappalysin-1	Q13219	**ECM Regulators**	2.74	none (1.51×10^-1^)	1.94	**increase (2.24×10**^**-2**^**)**
*PCOLCE*	Procollagen C-endopeptidase enhancer 1	Q15113	ECM Glycoproteins	5.82	**increase (1.94×10**^**-5**^**)**	3.74	**increase (1.91×10**^**-3**^**)**
*PCOLCE2*	Procollagen C-endopeptidase enhancer 2	Q9UKZ9	ECM Glycoproteins	2.10	none (6.02×10^-2^)	1.78	**increase (4.76×10**^**-2**^**)**
*PPBP*	Platelet basic protein	P02775	**Secreted Factors**	8.06	**increase (1.62×10**^**-4**^**)**	5.28	**increase (6.31×10**^**-4**^**)**
*PRELP*	Prolargin	P51888	Proteoglycans	1.41	**increase (7.40×10**^**-3**^**)**	0.84	none (3.97×10^-2^)
*PRG4*	Proteoglycan 4	Q92954	Proteoglycans	3.38	**increase (9.58×10**^**-3**^**)**	3.16	none (8.86×10^-2^)
*S100A11*	Protein S100-A11	P31949	**Secreted Factors**	-1.87	none (2.59×10^-1^)	-3.42	**decrease (8.23×10**^**-2**^**)**
*S100A13*	Protein S100-A13	Q99584	**Secreted Factors**	4.73	**increase (1.05×10**^**-2**^**)**	3.32	**increase (3.00×10**^**-2**^**)**
*S100A4*	Protein S100-A4	P26447	**Secreted Factors**	-0.46	none (4.18×10^-1^)	-1.22	**decrease (1.68×10**^**-2**^**)**
*SBSPON*	Somatomedin-B and thrombospondin type-1 domain-containing protein	Q8IVN8	ECM Glycoproteins	-0.23	none (7.09×10^-1^)	-1.48	**decrease (7.89×10**^**-2**^**)**
*SCUBE3*	Signal peptide, CUB and EGF-like domain-containing protein 3	Q8IX30	**Secreted Factors**	2.36	**increase (1.94×10**^**-2**^**)**	1.26	**increase (4.07×10**^**-2**^**)**
*SERPINA1*	Alpha-1-antitrypsin	P01009	**ECM Regulators**	0.11	none (8.44×10^-1^)	-1.74	**decrease (4.74×10**^**-3**^**)**
*SERPINA10*	Protein Z-dependent protease inhibitor	Q9UK55	**ECM Regulators**	4.04	**increase (4.77×10**^**-4**^**)**	3.79	**increase (1.33×10**^**-2**^**)**
*SERPINA3*	Alpha-1-antichymotrypsin	P01011	**ECM Regulators**	-0.18	none (6.34×10^-1^)	-1.20	**decrease (4.03×10**^**-3**^**)**
*SERPINA4*	Kallistatin	P29622	**ECM Regulators**	1.09	**increase (1.87×10**^**-2**^**)**	1.14	**increase (7.40×10**^**-3**^**)**
*SERPINA5*	Plasma serine protease inhibitor	P05154	**ECM Regulators**	3.38	**increase (5.19×10**^**-4**^**)**	2.93	**increase (4.95×10**^**-3**^**)**
*SERPINB9*	Serpin B9	P50453	**ECM Regulators**	-0.30	none (8.06×10^-1^)	-2.87	**decrease (4.01×10**^**-2**^**)**
*SERPINC1*	Antithrombin-III	P01008	**ECM Regulators**	2.23	**increase (1.32×10**^**-3**^**)**	1.44	**increase (9.90×10**^**-3**^**)**
*SERPINE1*	Plasminogen activator inhibitor 1	P05121	**ECM Regulators**	1.61	none (1.31×10^-1^)	2.08	**increase (2.06×10**^**-2**^**)**
*SERPINE2*	Glia-derived nexin	P07093	**ECM Regulators**	1.37	none (6.99×10^-2^)	1.09	**increase (1.40×10**^**-2**^**)**
*SERPING1*	Plasma protease C1 inhibitor	P05155	**ECM Regulators**	-1.09	**decrease (1.48×10**^**-2**^**)**	-1.35	**decrease (7.45×10**^**-4**^**)**
*SERPINH1*	Serpin H1	P50454	**ECM Regulators**	1.25	**increase (1.13×10**^**-3**^**)**	0.30	none (8.50×10^-2^)
*SFRP4*	Secreted frizzled-related protein 4	Q6FHJ7	**Secreted Factors**	4.80	none (5.72×10^-2^)	3.28	**increase (1.06×10**^**-2**^**)**
*SLIT2*	Slit homolog 2 protein	O94813	ECM Glycoproteins	1.62	none (1.74×10^-1^)	4.10	**increase (4.72×10**^**-2**^**)**
*SPARC*	SPARC	P09486	ECM Glycoproteins	-5.68	**decrease (3.72×10**^**-3**^**)**	-5.62	**decrease (9.43×10**^**-3**^**)**
*SPARCL1*	SPARC-like protein 1	Q14515	ECM Glycoproteins	-5.92	**decrease (6.77×10**^**-4**^**)**	-4.51	**decrease (5.73×10**^**-3**^**)**
*SPON1*	Spondin-1	Q9HCB6	ECM Glycoproteins	2.95	none (1.45×10^-1^)	2.21	**increase (6.05×10**^**-2**^**)**
*TGFBI*	Transforming growth factor-beta-induced protein ig-h3	Q15582	ECM Glycoproteins	1.60	**increase (3.27×10**^**-2**^**)**	0.64	none (7.91×10^-3^)
*THBS4*	Thrombospondin-4	P35443	ECM Glycoproteins	6.98	**increase (5.42×10**^**-3**^**)**	2.13	**increase (3.62×10**^**-2**^**)**
*TIMP1*	Metalloproteinase inhibitor 1	P01033	**ECM Regulators**	7.97	**increase (1.02×10**^**-5**^**)**	4.02	**increase (1.40×10**^**-4**^**)**
*TIMP2*	Metalloproteinase inhibitor 2	P16035	**ECM Regulators**	9.69	**increase (1.69×10**^**-2**^**)**	3.82	**increase (2.21×10**^**-3**^**)**
*TIMP3*	Metalloproteinase inhibitor 3	P35625	**ECM Regulators**	1.94	none (1.73×10^-1^)	1.18	**increase (4.84×10**^**-2**^**)**
*TINAGL1*	Tubulointerstitial nephritis antigen-like	Q9GZM7	ECM Glycoproteins	-1.03	none (1.15×10^-1^)	-1.05	**decrease (3.32×10**^**-2**^**)**
*TNXB*	Tenascin-X	P22105	ECM Glycoproteins	2.44	**increase (4.01×10**^**-3**^**)**	2.51	**increase (1.02×10**^**-3**^**)**
*VCAN*	Versican core protein	P13611	Proteoglycans	-4.29	**decrease (9.32×10**^**-5**^**)**	-3.79	**decrease (2.97×10**^**-4**^**)**

We identified 16 serine protease inhibitor proteins (SERPINs) in the CEX peaks, of which 5 and 6 had a significantly higher abundance compared to the AEX peaks and the protein extracts, respectively. Abundance of several proteoglycans including aggrecan, versican, biglycan, lumican and decorin, significantly decreased in the CEX peaks, compared to both the protein extracts and AEX peaks (Fig. 7A, B and Table 4).

**Fig. 7:**
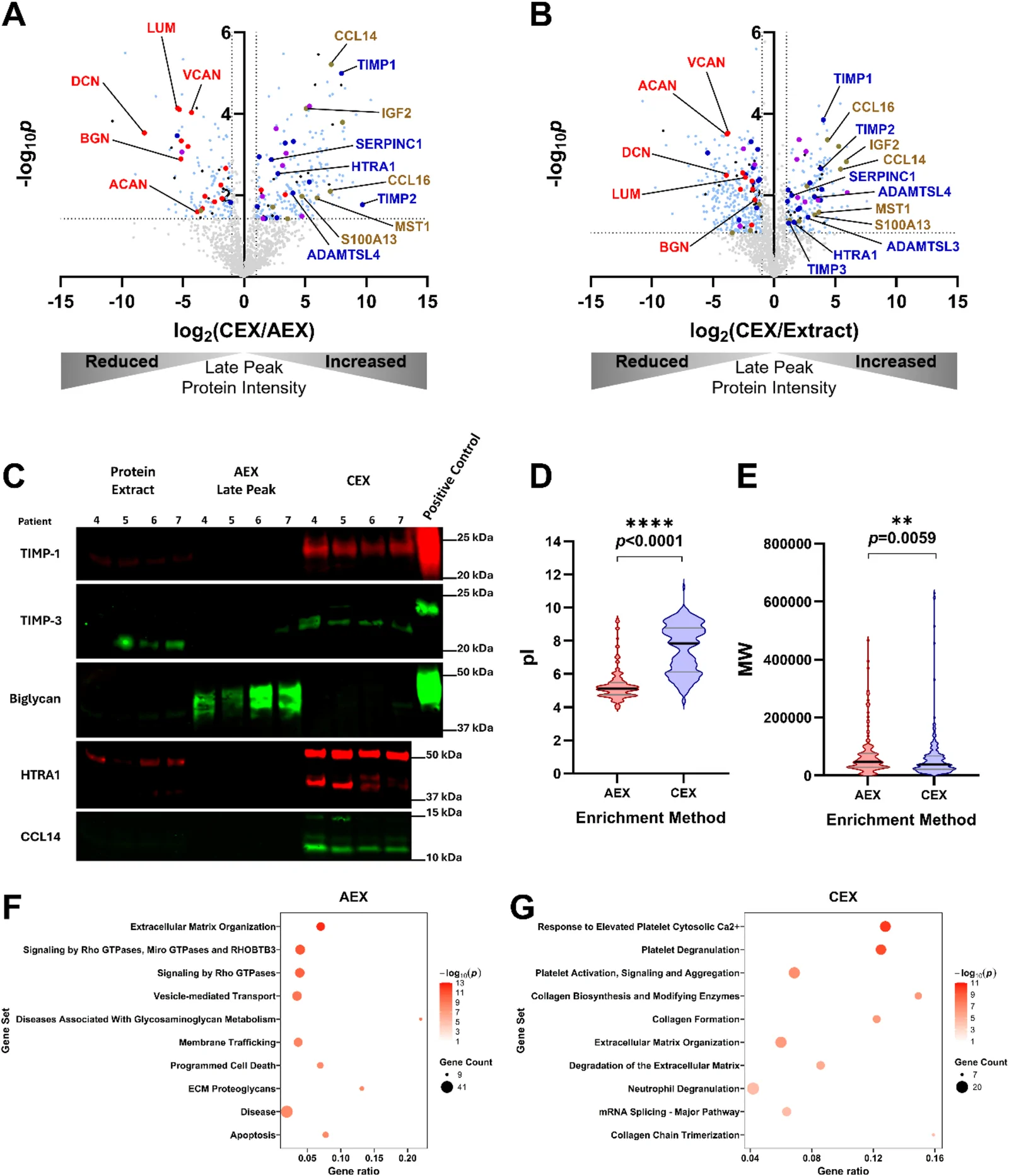
**AEX and CEX show selective enrichment of proteoglycans and ECM regulators/secreted factors, respectively. A, B)** Volcano plots comparing CEX peaks with A) AEX late peaks and B) protein extracts. Grey points: non-significantly changed proteins; light blue points: significantly increased/decreased non-ECM proteins; large red points: significantly increased/decreased proteoglycans; large gold points: significantly increased/decreased ECM secreted factors; large dark blue points: significantly increased/decreased ECM regulators; large purple points: significantly increased/decreased collagens; black points: other significantly increased/decreased matrisome proteins. Dashed lines indicate significance thresholds. **C)** Immunoblots of TIMP-1, TIMP-3, biglycan, HTRA1 and CCL14. Numbers above lanes indicate different patients. MW, molecular weight. **D, E)** Violin plots showing the distributions of D) isoelectric points (pI) and E) molecular weights (MW) for AEX and CEX peaks; **: *p <* 0.01, ****: *p <* 0.0001 by the Kolmogorov-Smirnov test. **F, G)** Gene set analyses, as per the Reactome database showing the 10 most significantly represented gene sets, as per adjusted *p*-values, for the significant proteins identified in A. Circle sizes correspond to total gene counts. (For interpretation of the references to colour in this figure legend, the reader is referred to the web version of this article.)

The increased abundance of HTRA1, TIMP-1, TIMP-3 and CCL14 and decreased abundance of biglycan in CEX peaks was orthogonally confirmed by immunoblot analysis (Fig. 7C). Compared to AEX, proteins more abundant in CEX had a significantly higher pI (median value:7.84 [Q_1_ 6.12, Q_3_ 8.77] compared to 5.12 [Q_1_ 4.74, Q_3_ 5.48]) and lower MW (median value: 37.7 kDa [Q_1_ 21.7, Q_3_ 68.0], compared to 47.5 kDa [Q_1_ 28.3 kDa, Q_3_ 76.1 kDa]) (Fig. 7D and E).

Functional enrichment analysis of the differentially abundant proteins in CEX compared to AEX correlated to an upregulation of pathways related to *“Collagen biosynthesis and modification, formation and chain trimerization”* and *“Degradation of the ECM”. “ECM organization”* was a shared significantly represented gene set, with AEX showing highly expressed *“ECM proteoglycans”* and *“Diseases associated with GAG metabolism”* gene sets (Fig. 7F and G). Overall, these results highlight an enrichment of ECM regulators, in particular proteases, protease inhibitors and other ECM-modifying enzymes, and secreted factors in the CEX fraction, and an increase of proteoglycan-related gene sets in the AEX fraction.

## Discussion

Proteoglycans and matrisome-associated proteins, in particular ECM regulators and secreted factors, present distinct challenges for detection by LC-MS/MS. The large aggregating proteoglycans/hyalectans aggrecan and versican have a low density of tryptic sites, making fragmentation and subsequent mass spectrometric analysis inefficient. Moreover, because of their high density of GAG-attachment sites, the resulting tryptic peptides may be poorly ionized and give fewer matches due to variable mass modification by glycosylation [11]. In contrast, ECM regulators, in particular proteases and protease inhibitors, and secreted factors, have lower abundance and MW [18], [19], limiting their detection at the peptide level in LC-MS/MS due to masking by more abundant proteins. In tandem, these factors cause poor identification and peptide coverage for these matrisome components. Here, we addressed some of these challenges by expanding on previous work on aortic tissues [5], [26].

Using LC-MS/MS, we demonstrated that, although Gu-HCl is effective in extracting proteoglycans, ECM regulators and secreted factors from ECM-rich tissues such as the aorta, sequential ion-exchange chromatography results in the enrichment of these matrisome classes. This is particularly relevant for their downstream, non-MS based functional and biological characterization. Compared to Gu-HCl, alternative extraction methods are less suitable for characterizing proteoglycans and ECM regulators. Urea, for example, decomposes into toxic cyanate [31]. Direct sonication into sodium dodecyl sulphate (SDS) also solubilizes aortic cytoplasmic and nuclear proteins [26], [32] and is therefore less efficient in extracting highly cross-linked or core matrisome components [33], [34]. Addition of a 0.5 M NaCl extraction step has been shown to release loosely bound ECM associated proteins and ECM protein fragments, but does not specifically enrich in ECM regulators or secreted factors [26].

Initially, we provided experimental evidence supporting AEX chromatography as a selective proteoglycan enrichment approach. Among the 26 proteoglycans identified, versican, aggrecan, biglycan, decorin and lumican were detected in previous proteomic studies of human aneurysmal [5] and non-aneurysmal [26] aortas. Versican and biglycan were also recently identified in an N-terminomic study of aneurysmal aortic tissues extracted in Gu-HCl [35].

Versican is a well-characterized component of the aortic ECM, being predominantly expressed in the media layer [36]. Aggrecan is the major proteoglycan in cartilage, but its presence in the aortic wall and other vascular beds has recently emerged [37]. Both aggrecan and versican accumulate in human TAA [5]. The SLRPs decorin, biglycan and lumican play a role in the assembly of fibrillar collagens [38]. Reduced levels of decorin and biglycan have been implicated in TAAD [7], [8], [39]. Increased serum lumican levels have been proposed as a biomarker of TAAD [40], [41].

For the hyalectans aggrecan and versican, the central GAG-rich regions represent a formidable challenge to MS detection. Factors including peptides outside the defined mass window and unfavorable charge status due to the presence of GAGs [11] may explain why no significant changes in overall versican or aggrecan abundances were observed in the AEX fractions, despite their heavy glycosylation conferring the ability to bind AEX resin with high affinity. Nevertheless, for both hyalectans, PLF analysis indicated increased intensities of C-terminal peptides in the AEX late peaks, concurrent with significantly decreased intensities of N-terminal peptides compared to protein extracts. Immunoblot analysis showed that AEX late peaks retained high MW species while losing lower MW species such as versikine, as the NaCl gradient effectively separated proteoglycan fragments from full-length proteoglycans.

Similarly, our PLF analysis showed that peptide intensity for biglycan and lumican was significantly higher in the AEX late peaks compared to AEX early peaks across large portions of their sequences, particularly in the leucine-rich repeats, indicating retention of more intact species, in agreement with immunoblot analysis. This is an example of how PLF combined with orthogonal data can shed light on proteolytic remodeling and, as far as we know, the first application of PLF to proteomic studies of the human aorta.

Matching ex vivo semi-tryptic peptides, i.e., peptides with a single tryptic terminus originating from tissue extracts, with proteoglycan neopeptides identified in vitro upon digestion of isolated proteoglycans/protein extracts with individual proteases [10], [35] can provide indications of endogenous proteolytic activity where proteases themselves are not directly detectable. Indeed, PLF can identify the key locations of sequence variation and even proteolytic susceptibility of a variety of ECM proteins [23], [24], [25] and can be combined with emerging degradomic techniques [42], [43] for prediction of endogenous proteolysis.

In addition to TAA, proteolytic ECM remodeling is widely implicated in disease and development, with dysregulation of the protease/inhibitor ratio being a key feature of cancer, degenerative joint diseases, fibrosis and more [44]. Yet, both proteases and their inhibitors are difficult to extract and characterize from tissues due to their low abundance, short half-life and ECM association [19]. Similar limitations apply to growth factors, morphogens and cytokines [45]. Our workflow leverages the different distribution of pIs between ECM regulators and proteoglycans by subjecting the unbound fraction from AEX to an additional step of CEX chromatography. While a heparin Sepharose CEX-based approach has recently been applied to cell media [46] and human bodily fluids [47], and has historically been used to isolate growth factors from human tissues [48], this is, to our knowledge, its first coupling to AEX as an additional fractionation step as well as its first proteomic application to human tissues.

CEX chromatography achieved a selective enrichment in ECM regulators (4.4% of the CEX proteome) and identified 53 proteins falling within this category, including the metalloprotease inhibitors TIMP-1, TIMP-2, and TIMP-3, the serine protease inhibitor SERPINC1/antithrombin III, ADAMTSL3 (an ADAMTS lacking catalytic activity which regulates TGFβ signaling in cardiovascular tissues) [49], ADAMTSL4, which regulates microfibril biogenesis and interacts with lysyl oxidases [50], and the serine protease HTRA1. TIMPs are endogenous inhibitors of matrix metalloproteinases, ADAMS, and ADAMTSs [51]. TIMP-3 has strong inhibitory activity against ADAMTS1 [52], ADAMTS4, ADAMTS5 [53], and ADAMTS9 [11], all proteases with reported activity against the aortic proteoglycan versican [11], [53], [54]. Notably, although these ADAMTSs were not directly detected in CEX peaks, their proteolytic signature (versikine) was abundant in protein extracts and AEX early peaks. Among the secreted factors we identified in CEX peaks, circulating levels of CCL14 were recently found to be strongly associated with single nucleotide polymorphisms associated with spontaneous coronary artery dissection in the UK Biobank **[55]**.

Many ECM proteins are known to bind to heparin [29]. The consensus motif for interaction with heparin is relatively loose (−X-B-B-X-B-X- and -X-B-B-B-X-X-B-X- where Bs are basic residues and X is a hydrophobic residue) [56], but some proteins, like antithrombin III, have very strict requirements in terms of HS sulfation [30]. It remains to be confirmed by direct binding studies if the proteins we identified in CEX peaks constitute bona fide heparin ligands.

Our study provides several strengths and advances for tissue-based ECM research. Firstly, our workflow improves enrichment of proteoglycans, ECM regulators and secreted factors, as reflected in 4 key observations: 1) improved sequence coverage of multiple proteoglycans in AEX fractions compared with protein extracts; 2) significantly increased abundance of proteoglycans, in particular SLRPs, in AEX fractions compared with protein extracts; 3) greater representation of proteoglycans in the top ranked proteins in AEX peaks; 4) enhanced detectability of proteoglycans, ECM regulators and secreted factors by orthogonal methods. Notably, the advantages of AEX/CEX enrichment were evident not only by LC-MS/MS but also by immunoblotting, suggesting that the increased representation of proteoglycans, ECM regulators and secreted factors achieved by this workflow may benefit downstream analytical approaches commonly employed in ECM research outside of bottom-up proteomics and immunoblot. Although advances in mass spectrometry instrumentation continue to improve sensitivity and proteome coverage **[57]**, the benefits of AEX/CEX enrichment derive from biochemical fractionation rather than instrument performance alone. By reducing sample complexity and increasing the relative abundance of proteoglycans, ECM regulators and secreted factors, our workflow improves the representation and characterization of these challenging ECM components prior to LC-MS/MS analysis. Consequently, while newer instruments would be expected to increase the total number of proteins and peptides identified, the relative enrichment of these specific matrisome components should remain across different analytical platforms.

Secondly, our workflow also provides the first map of the human aortic heparin ligandome (Supplementary file 1) and can be potentially applied to identify differences between TAA and non-TAA controls and dissect disease mechanisms.

Nevertheless, our approach presents some limitations. As it involves enrichment and fractionation it has low sample throughput, limiting its application in clinical settings, and to large populations. However, improvements in multidimensional liquid chromatography coupled with mass spectrometry, including those using ion exchange chromatography **[58]**, may lessen researchers' workload and substantially increase throughput. The need for substantial amounts of wet tissue (≥400 mg) is another limitation, although this is likely tissue-dependent (i.e., different proportions of ECM versus non-ECM material). The advent of convenient data-independent acquisition methods for LC-MS/MS [59] and more sensitive mass spectrometers are rapidly enabling the detection of low abundance proteins (like ECM regulators and secreted factors) within a complex background (without the need for enrichment) compared to data-dependent acquisition (DDA)-centered approaches. How our pipeline performs with these new analytical capabilities requires further investigation. Moreover, our proteo/glycanome data represents multiple cell types, so single-cell proteomics would be required to differentiate cellular subtypes. Finally, from a translational perspective, our studies analyzed only a limited number of patients, so the data may not be representative of bigger, more heterogenous populations. However, the aim of this study was to establish and validate an analytical workflow, while future larger studies will compare TAA tissues with those of appropriate non-TAA controls.

In conclusion, we presented a workflow for analysis of tissue extracts, which successfully enriched challenging-to-characterize components of the ECM and increased their detection. We confirmed enrichment of proteoglycan content in AEX chromatography and provided a novel approach for the enrichment of ECM regulators including proteases and protease inhibitors as well as secreted factors. Peptides identified at high abundance with this method can then be prioritized for detection in both tissues and biological fluids, for both biomarker discovery and mechanistic studies.

## Methods

### Human samples

TAA tissues were obtained from patients undergoing elective surgical replacement of an aneurysmal ascending aorta and/or aortic root at the Royal Brompton, Harefield and Hammersmith Hospitals, London, UK. This study complied with the declaration of Helsinki. Ethical approval was granted by London - West London & GTAC Research Ethics (NHS REC study reference: 21/LO/0301). All subjects provided written informed consent and their participation in the research study did not cause any deviation from routine clinical practice or additional diagnostic/therapeutic procedures. Patient information is reported in Supplementary Table 1.

Tissues (1.5–2 g) were washed with phosphate buffered saline (PBS), pH 7.4 (Merck, P4417), dissected into small fragments (approximately 25 mm^2^ surface area), and flash-frozen in liquid nitrogen before being stored at -80 °C.

### Tissue homogenization and protein extraction

Samples were mechanically homogenized and placed into protein extraction buffer (*4 M guanidine hydrochloride (Gu-HCl), 2% w/v 3-[(3-Cholamidopropyl)dimethylammonio]-1-propanesulfonate, 50 mM sodium acetate, 20 mM ethylenediaminetetraacetic acid,* pH 5.8–6.0), supplemented with protease inhibitor cocktail (1:200, APExBIO, K1008) at a ratio of 100 mg tissue: 1 mL extraction buffer, and gently mixed for 48–72 h at 4 °C. Samples were then centrifuged at 35,000 x*g* for 1 h at 4 °C. The supernatant was collected, filtered (0.45 μm) to remove any residual insoluble fraction, and dialyzed extensively into Buffer A (*20 mM HEPES, 150 mM NaCl, pH 7.5*). Aliquots of dialyzed extracts were taken for analysis.

### AEX and CEX chromatography

Chromatography was carried out using an Åkta Start system (GE Healthcare). For AEX, dialyzed extracts were loaded onto a 5 mL DEAE Sepharose column (HiTrap FF, Cytiva 17515401). The column was washed extensively with Buffer A (10 column volumes, CV) and bound material was eluted on a gradient over 10 CV of 0–100% – Buffer B (*20 mM HEPES, 1 M NaCl, pH 7.5*). The column was regenerated by washing with 10 CV 2 M NaCl. Protein content monitored by measuring A_280_.

For CEX chromatography, the unbound fraction of AEX was loaded onto a 1 mL Heparin Sepharose column (HiTrap HP, Cytiva 17040601). The column was washed with 10 CV of TBS Buffer (*20 mM Tris, 150 mM NaCl, pH 7.5*) and bound material was eluted on a gradient over 10 CV of 0–100% TBS Elution Buffer (*20 mM Tris, 2 M NaCl, pH 7.5*).

### Dimethylmethylene blue (DMMB) assay

To identify the presence and relative amounts of sulphated GAGs, eluted AEX fractions were mixed 1:10 with DMMB solution (*16 mg/L DMMB, 3.2 g/L glycine, 0.4 g/L NaCl, 10 mM acetic acid),* as previously described [60]. Absorbance was measured at 525 nm on a Spectramax i3x plate reader (Molecular Devices).

### Deglycosylation

Fractions corresponding to AEX and CEX early and late peaks were pooled, concentrated 20-fold using 3 kDa cut-off centrifugal devices (Millipore UFC9003) and dialyzed back into Buffer A or TBS to ensure consistent NaCl concentration. Protein concentrations were determined by Pierce™ BCA Protein Assays (ThermoFisher, 23225) and validated by SDS-PAGE followed by Coomassie Brilliant Blue staining (Supplementary Fig. 11 and 12). Samples were deglycosylated at a ratio of 150 μU enzyme/μg protein with chondroitinase ABC (AMSbio AMS.E1028–02), PNGase F (Merck, P7367), *endo*-β-galactosidase/keratanase (Merck, G6920), and heparinase II (Merck, H6512) in deglycosylation buffer (*25 mM sodium acetate, 12.5 mM Tris pH 8.0, 12.5 mM ethylenediaminetetraacetic acid*) for 16 h at 37 °C **[26]**.

### SDS-PAGE and immunoblot analysis

Deglycosylation reactions were stopped by adding 4× NuPAGE™ LDS sample buffer (ThermoFisher NP0008) containing 5% β-mercaptoethanol and incubated at 95 °C for 10 min. Samples and positive controls where available (Supplementary Table 2) were then subjected to SDS-PAGE at 200 V for 45–60 min (depending on the MW of the protein of interest) on 4–12% Bis-Tris Mini Protein Gels (Invitrogen, NP0323). Proteins were transferred to a nitrocellulose membrane via a semi-dry transfer method using a Trans-Blot Turbo Transfer System (BioRad, 25 V, 30 min), and membranes were placed in 3% bovine serum albumin (BSA)/PBS before incubation with an appropriate antibody (all in 0.5%BSA/PBS) (Supplementary Table 2) at 4 °C overnight. Membranes were then washed 3 times with PBS containing 0.1% TWEEN-20 (PBS-T) for 10 min each, before being placed in the appropriate fluorescent- labelled secondary antibody solutions (LiCor IRDye®, 1:5000 in 0.5% *w*/*v* BSA/PBS) or horseradish peroxidase-conjugated secondary antibody (1:10,000 in 0.5% *w*/*v* BSA/PBS) (Agilent, P0447) for one hour, after which there was a repeat of the PBS-T washes. Membranes were then imaged on a LiCor Odyssey Imager, at the appropriate 680 nm, 800 nm or chemiluminescent wavelength. Unedited blots are shown in Supplementary Fig. 13–24.

### LC-MS sample preparation

Protein from deglycosylated samples was isolated onto S-Trap™ Mini columns (Protifi, C02-micro-80) as per manufacturer's instructions, before addition of Trypsin Gold (Promega, V5280), dissolved in digestion buffer (*50 mM triethylammonium bicarbonate*), at a 1:10 trypsin: protein ratio. Where indicated, GluC (Promega, V1651) was added instead of trypsin at a 1:20 GluC/protein ratio. All digestions were carried out overnight at 37 °C. The columns were then washed with digestion buffer, 0.1% *v*/v formic acid, and finally 30% *v*/v acetonitrile to elute trypsinized peptides. Peptides were desalted using OLIGO™ R3 Reversed - Phase Resin (ThermoFisher, 1133903), and a 0.45 μm filter plate (Cytiva, 8582). Peptides were loaded onto the resin and mixed before washing with 0.1% formic acid and finally eluted with 30% acetonitrile in 0.1% formic acid, before lyophilization in a vacuum centrifuge.

### LC-MS/MS

Mass Spectrometry data was obtained on a Lumos™ Fusion™ Orbitrap mass spectrometer (Thermo). Samples (2 μL) were injected into a nano-HPLC column (*Waters nanoEase M/Z Peptide CSH C18 Column, 130 Å, 1.7* μ*m, 75* μ*m × 250 mm)* at 35 °C, 300 nL/min. Multistage gradients of increasing acetonitrile concentration (0–80%), dissolved in 0.1% formic acid, were used to separate tryptic peptides, over a 60-min run, with a total method time of 75 min. Samples were passed over a Nanospray Flex ion source (ThermoFisher) set at 2,000 V with a chamber temperature of 275^o^ C. Peptides were selected for fragmentation by a DDA approach.

The MS proteomics data have been deposited to the ProteomeXchange Consortium via the PRIDE [61] partner repository with the dataset identifier PXD078737.

All proteins and peptides identified are provided in Supplementary files 2–3 (proteins) and **4–5** (peptides).

### Data analysis

Spectral data were analyzed on MaxQuant [62], using the following parameters: semi-specific digestion, minimum peptide length 7 amino acids, fixed modifications of carbamidomethylation, variable modifications of oxidation (methionine) and acetylation, and DDA label-free quantification with the match between runs setting on. False discovery rates (FDRs) were set at 0.01 for AEX “proteoglycanome” data (Fig. 2, Fig. 3, Fig. 5), due to the importance of individual peptides for analysis, and at 0.05 for CEX vs AEX “heparin ligandome” data (Fig. 6, Fig. 7). “Proteoglycanome” data was additionally searched with MaxQuant's variable Hex(1)HexNAc(1) (ST) glycosylation modification. “Heparin Ligandome” data was searched with fully-specific digestion and with proline oxidation as a variable modification. Data were further processed using Perseus **[63]** to remove contaminants, reverse hits and site-specific identification. Only proteins present in at least *n-1* samples in at least one condition were considered for all downstream analysis and only proteins present in at least *n-1* samples were subsequently considered as present in each fraction. For the proteoglycanome dataset, 1,010 proteins were detected in all 3 fractions (protein extract, AEX early peak and AEX late peak), spanning an abundance range of >5 orders of magnitude. The median coefficient of variation of all quantified proteins, from the protein extract, was 33.4% between patients. For the heparin ligandome data, 840 common proteins were identified between protein extracts, AEX, and CEX fractions, spanning an abundance range of >4 orders of magnitude. Total sequence coverage from combined GluC/trypsin digests was computed from significantly identified peptide sequences using a custom R (Version 4.6.1) script. Peptide coordinates were mapped to the corresponding UniProt protein sequence, and the percentage of covered amino acid residues was determined for each protein.

To generate volcano and PCA plots, all missing intensity values for proteins were imputed after log_2_-transformation using the impute.qrilc function of the R package imputeLCMD, accounting for the right-skewed nature of our data. Significantly increased proteins were identified using a paired Student's *t*-test (*p* < 0.05) for all comparisons except for the AEX early vs late peaks and AEX late peaks vs CEX peaks, which used an unpaired Welch's *t*-test, and corrected using Perseus' in-built permutation-based FDR method (250 randomizations, FDR < 0.05, S_0_ = 0), requiring a > 2-fold change between conditions. All proteoglycans were defined using previous experimental glycosite validation [4]. Other ECM proteins were defined and categorized as per the MatrisomeDB [17]. Only proteins attributed to a single gene are reported.

pIs and MW were identified using the Expasy compute pI/MW tool [64]. Significantly increased proteins, as defined above, from each condition were measured, and significant differences in their pI and MW distributions were defined using the Kolmogorov-Smirnov test.

### Peptide location fingerprinting

Previously described PLF protocols [23], [24] were used to account for changes in peptide intensity [28] across the protein's sequence, including all identified peptides, dividing the sequence into predefined bins of fixed lengths (20 or 50 amino acids). All peptide intensities present in at least 2/3 replicates of at least one condition were considered for analysis. Here, we report fold changes between bin intensities, to account for large variations between conditions, instead of differences between bin intensities as previously published [28]. Statistical significance was determined by a 2-way repeated measures ANOVA between bin intensities of the late peak and protein extract, per protein, before a Bonferroni correction for multiple comparisons was applied.

### Enrichment analysis

Significant functional gene sets for each condition were identified using the Enrichr tool [65]. Gene names of significantly increased proteins from each condition were input into Enrichr, before being subjected to the software's Benjamini-Hochberg corrected Fisher exact test to identify the 10 most significant gene sets as determined by the Reactome database [66]. Adjusted *p*-values and gene ratios of significantly varied genes in the entire set were reported.

## CRediT authorship contribution statement

**Alexander F. Minns:** Writing – review & editing, Writing – original draft, Visualization, Validation, Methodology, Investigation, Formal analysis. **Ren Jie Tuieng:** Validation, Investigation, Formal analysis, Writing – review & editing. **Marsioleda Kemberi:** Investigation. **Yousuf Salmasi:** Resources. **Thanos Athanasiou:** Resources. **Anthony D. Whetton:** Writing – review & editing, Supervision. **Alexander Eckersley:** Writing – review & editing, Validation, Supervision, Methodology, Conceptualization. **Michael J. Sherratt:** Writing – review & editing, Supervision, Methodology, Funding acquisition, Conceptualization. **Salvatore Santamaria:** Writing – review & editing, Writing – original draft, Visualization, Validation, Supervision, Project administration, Methodology, Investigation, Funding acquisition, Formal analysis, Conceptualization.

## Ethics declaration

Written informed consent to take part in the study and to publish the article has been obtained from all participants or their legal representatives. The privacy rights of participants have been observed.

This study included organ or tissue donors.

This study includes human biological material and consent was obtained by donors, or their next of kin or legal representatives, for use in this study and for publication of the article. The samples used in this research were not sourced from executed prisoners or prisoners of conscience.

This study was performed in compliance with relevant laws, regulatory frameworks and guidelines where the research took place.

This study was approved by the NHS Health Research Autority-London - West London & GTAC Research Ethics Committee.

(Approval No. 21/LO/0301)

## Funding

This work was supported by an Intermediate Basic Science Research Fellowship from the 10.13039/501100000274British Heart Foundation (FS/IBSRF/20/25032, PI: SS), ECMAge Network Pump Priming Award (PPR104, PI: SS) and ECMAge Mobility award (E&M/R2/01, PIs: AFM and SS). AE is supported by the 10.13039/501100000268BBSRC (BB/Z517355/1). MJS and AE are supported by a programme grant from No7 Beauty Company (The Boots Group).

## Declaration of competing interest

The authors declare that they have no known competing financial interests or personal relationships that could have appeared to influence the work reported in this paper.

## Data Availability

Data will be made available on request.

## Appendix A Supplementary data

Supplementary Information

Supplementary File 1: Aortic heparin ligandome: all proteins identified in the CEX fraction

Supplementary File 2: List of all proteins identified in at least 2 replicates in at least one condition of the AEX material and protein extract comparison dataset

Supplementary File 3: List of all proteins identified in at least 3 replicates in at least one condition of the CEX material, AEX material and protein extract comparison dataset

Supplementary File 4: List of all peptides identified in at least 2 replicates in at least one condition of the AEX material and protein extract comparison dataset

Supplementary File 5: List of all peptides identified in at least 3 replicates in at least one condition of the CEX material, AEX material and protein extract comparison dataset
