# Supplementary material for: An analytical workflow enabling selective enrichment of proteoglycans and heparin ligands from the human aorta
Source: Matrix Biol Plus. 2026 Nov 1;32:100207. doi: 10.1016/j.mbplus.2026.100207 (PMC13769062; doi:10.1016/j.mbplus.2026.100207)
Supplement: Supplementary file 1 [file mmc1.docx]

**SUPPLEMENTARY INFORMATION**

**An analytical workflow enabling selective enrichment of proteoglycans and heparin ligands from the human aorta**

Alexander F. Minns,^1,2^ Ren Jie Tuieng,^3,4^, Marsioleda Kemberi,^2,5^ Yousuf Salmasi,^6^ Thanos Athanasiou,^6^ Anthony D. Whetton,^7^ Alexander Eckersley,^8,9^ Michael J. Sherratt,^3,9^ Salvatore Santamaria^1,2^*

^1^Discipline of Clinical Sciences, School of Biosciences, Faculty of Health and Medical Sciences, University of Surrey, Guildford, Surrey, United Kingdom;

^2^Department of Immunology and Inflammation, Imperial College London, London, United Kingdom;

^3^Division of Cell Matrix Biology & Regenerative Medicine, School of Biological Sciences, Faculty of Biology, Medicine and Health, The University of Manchester, Manchester, United Kingdom;

^4^Singapore Nuclear Research and Safety Institute, National University of Singapore, Singapore;

^5^Barts Health NHS Trust, The Royal Hospital, London, United Kingdom;

^6^Department of Surgery and Cancer, Faculty of Medicine, Imperial College London, London, United Kingdom;

^7^Veterinary Health Innovation Engine, Schools of Veterinary Medicine and Biosciences, University of Surrey, Guildford, Surrey, United Kingdom;

^8^Division of Musculoskeletal & Dermatological Sciences, School of Biological Science, Faculty of Biology, Medicine and Health, The University of Manchester, Manchester, United Kingdom;

^9^Manchester Cell Matrix Centre, School of Biological Science, Faculty of Biology, Medicine and Health, The University of Manchester, Manchester, United Kingdom;

* to whom the correspondence should be addressed: Salvatore Santamaria, ^1^Discipline of Clinical Sciences, School of Biosciences, Faculty of Health and Medical Sciences, University of Surrey, Guildford, Surrey, United Kingdom, [s.santamaria@surrey.ac.uk](mailto:s.santamaria@surrey.ac.uk); Department of Immunology and Inflammation, Imperial College London, London, United Kingdom, [s.santamaria@imperial.ac.uk](mailto:s.santamaria@imperial.ac.uk)

**This Supplementary Information contains Supplementary Figures 1-24 and Supplementary Tables 1-2**

**Supplementary Figure 1.** Principal component analysis of crude extract, early peak, and late peak fractions, n=3 patients.

**Supplementary Figure 2.** Venn diagrams showing protein identifications across trypsin-digested crude extracts, AEX early peaks and AEX late peaks.

**Supplementary Figure 3**. Peptide location fingerprinting analysis of biglycan showing overall intensities per bin. Significance was determined by a two-way repeated measures ANOVA, with Bonferroni-corrected multiple comparisons. *: p<0.05, **: p<0.01, ****: p<0.0001. Error bars show standard deviations.

**Supplementary Figure 4**. Peptide location fingerprinting analysis of lumican showing mean overall intensities per bin. Significance was determined by a two-way repeated measures ANOVA, with Bonferroni-corrected multiple comparisons. ****: p<0.0001. Error bars show standard deviations.

**Supplementary Figure 5**. Peptide location fingerprinting analysis of versican showing overall intensities per bin. White bars: protein extracts; red bars: AEX late peak. Significance was determined by a two-way repeated measures ANOVA, with Bonferroni-corrected multiple comparisons. ****: p<0.0001. Error bars show standard deviations.

**Supplementary Figure 6**. Peptide location fingerprinting analysis of aggrecan showing overall intensities per bin. White bars: protein extracts; red bars: AEX late peak. Significance was determined by a two-way repeated measures ANOVA, with Bonferroni-corrected multiple comparisons. ****: p<0.0001. Error bars show standard deviations.

**Supplementary Figure 7**. Peptide location fingerprinting analysis of decorin showing overall intensities per bin. Significance was determined by a two-way repeated measures ANOVA, with Bonferroni-corrected multiple comparisons. *: p<0.05, **: p<0.01, ****: p<0.0001. Error bars show standard deviations.

**Supplementary Figure 8.** A representative heparin Sepharose CEX chromatogram, showing UV absorbance over increasing sodium chloride concentrations.

**Supplementary Figure 9.** Principal component analysis of crude extract, AEX late peaks, and CEX peaks material. N=4 patients.

**Supplementary Figure 10.** Venn diagrams showing protein identifications across crude extracst, AEX late peaks and CEX peaks.

**Supplementary Figure 11.** Coomassie Brilliant Blue staining for samples run in immunoblots shown in **Figure 4** in the main text. MW, molecular weight.

**Supplementary Figure 12.** Coomassie Brilliant Blue staining for samples run in immunoblots shown in **Figure 6C** in the main text. MW, molecular weight.

**Supplementary Figure 13.** Full 2B6 immunoblot with yellow box corresponding to the area shown in **Figure 2B** in the main text.

**Supplementary Figure 14.** Full anti-biglycan immunoblot with yellow box corresponding to the area shown in **Figure 4A** in the main text.

**Supplementary Figure 15.** Full anti-decorin immunoblot with yellow box corresponding to the area shown in **Figure 4A** in the main text.

**Supplementary Figure 16.** Full 2B6 immunoblot with yellow box corresponding to the area shown in **Figure 4A** in the main text.

**Supplementary Figure 17.** Full anti-versican immunoblot with yellow box corresponding to the area shown in **Figure 4B** in the main text.

**Supplementary Figure 18.** Full anti-versikine immunoblot with yellow box corresponding to the area shown in **Figure 4B** in the main text.
**Supplementary Figure 19.** Full anti-aggrecan immunoblot with yellow box corresponding to the area shown in **Figure 4C** in the main text.

**Supplementary Figure 20.** Full anti-TIMP-1 immunoblot with yellow box corresponding to the area shown in **Figure 7C** in the main text.

**Supplementary Figure 21.** Full anti-TIMP-3 immunoblot with yellow box corresponding to the area shown in **Figure 7C** in the main text.

**Supplementary Figure 22.** Full anti-biglycan immunoblot with yellow box corresponding to the area shown in **Figure 7C** in the main text.

**Supplementary Figure 23.** Full anti-HTRA1 immunoblot with yellow box corresponding to the area shown in **Figure 7C** in the main text.

**Supplementary Figure 24.** Full anti-CCL14 immunoblot with yellow box corresponding to the area shown in **Figure 7C** in the main text.

**Supplementary Table 1.** Patient data from the aortic samples used in this study. Patient numbers refer to labels in the main text. TAA, thoracic aortic aneurysm; BAV, bicuspid aortic valve; TAV, tricuspid aortic valve.

| Dataset | Patient number | Age | Sex | Pathology | Valve morphology |
| --- | --- | --- | --- | --- | --- |
| AEX versus protein extract | 1 | 52 | M | Sporadic TAA | TAV |
|  | 2  3 | 56  71 | M  M | Sporadic TAA  Sporadic TAA | TAV  TAV |
| AEX versus CEX | 1  2 | 71  56 | M  F | Sporadic TAA  Sporadic TAA | TAV  TAV |
|  | 3 | 72 | F | Sporadic TAA | BAV |
|  | 4 | 79 | M | Sporadic TAA | BAV |

**Supplementary Table 2.** List of antibodies and positive controls used in this study. CS, chondroitin sulfate. N/A, not available. See main text for references.

| **Target** | **Positive Control**  **(Reference / Catalogue Number)** | **Host** | **Antibody Supplier**  **(Catalogue Number)** | **Antibody Registry ID** | **Dilution (Concentration)** |
| --- | --- | --- | --- | --- | --- |
| Aggrecan | Aggrecan from bovine nasal cartilage (gift from Dr Kazuhiro Yamamoto, University of Liverpool) | Mouse | Thermo Fisher Scientific (AHP0022) | AB_2536346 | 1:50 (20 µg/mL) |
| Biglycan | In-house recombinant human biglycan **[52]** | Goat | Abcam  (ab58562) | AB_940565 | 1:500  (1 µg/mL) |
| CCL14 | N/A | Rabbit | Thermo Fisher Scientific  (500-P106) | AB_2929218 | 1:1,000  (500 ng/mL) |
| CS | In-house recombinant human biglycan **[52]** | Mouse | AMSBio  (270432-CS, clone 2B6) | N/A | 1:100 (Serum) |
| Decorin | Recombinant human decorin  (Bio-Techne, 143-DE) | Mouse | Santa Cruz Biotechnology  (sc-73896) | AB_2090274 | 1:200  (500 ng/mL) |
| HTRA1 | N/A | Rabbit | Cell Signaling Technology (44783) | N/A | 1:1000  (530 ng/mL) |
| TIMP-1 | Recombinant human TIMP1  (Bio-Techne, 970-TM) | Goat | Bio-Techne  (AF970) | AB_355751 | 1:500 (2 µg/mL) |
| TIMP-3 | Recombinant human TIMP3  (Bio-Techne, 973-TM) | Rabbit | Sigma-Aldrich (Ab6000) | AB_10562965 | 1:500 (1 µg/mL) |
| Versican | In-house recombinant human versican **[54]** | Rabbit | Abcam  (ab270444-1001) | N/A | 1:1000  (625 ng/mL) |
| Versikine | In-house recombinant human versican v1 digested with ADAMTS5 **[54]** | Rabbit | Thermo Fisher Scientific  (PA11748A) | AB_2304324 | 1:1000  (2 µg/mL) |
